# Vitamin E TPGS based transferosomes augmented TAT as a promising delivery system for improved transdermal delivery of raloxifene

**DOI:** 10.1371/journal.pone.0226639

**Published:** 2019-12-27

**Authors:** Nabil A. Alhakamy, Usama A. Fahmy, Osama A. A. Ahmed

**Affiliations:** 1 Department of Pharmaceutics, Faculty of Pharmacy, King Abdulaziz University, Jeddah, Saudi Arabia; 2 Department of Pharmaceutics & Industrial Pharmacy, Faculty of Pharmacy, Minia University, Minia, Egypt; Peking University, CHINA

## Abstract

Raloxifene is commonly used for breast cancer protection. The low bioavailability of raloxifene (2%) is the result of its low solubility and intestinal glucuronidation. The nano-lipid carriers are characterized by small particle size, biocompatibility, and sustainable properties that improve cellular uptake of the loaded drug. The aim of this study was the improvement of raloxifene bioavailability by enhancing its solubility and cellular penetration through formulation of D-α-tocopheryl polyethylene glycol 1000 succinate based transferosomes and augmenting their effect with the cationic cell-penetrating peptide transactivator of transcription of the human immunodeficiency virus. Particle size, zeta potential, and transmission electron microscope investigation of the formed nanocarriers were carried out. *Ex vivo* raloxifene permeation through rat skin and cell viability studies was investigated. The results of D-α-tocopheryl polyethylene glycol 1000 succinate- transactivator of transcription of the human immunodeficiency virus transferosomes showed an average vesicle size of 96.05 nm with positively charged vesicles 39.4 mV of zeta potential value. The results revealed significant (*p* < 0.05) enhancement of raloxifene permeation from raloxifene transferosomes- loaded film when compared with raw raloxifene film. IC50 results showed significant improvement of formulated raloxifene cytotoxicity by 1.42-fold in comparison with raw raloxifene against MCF-7 cell lines. The developed raloxifene—transferosomes are considered promising nano-lipid carriers for the enhancement delivery of raloxifene.

## Introduction

**Fig 1:**
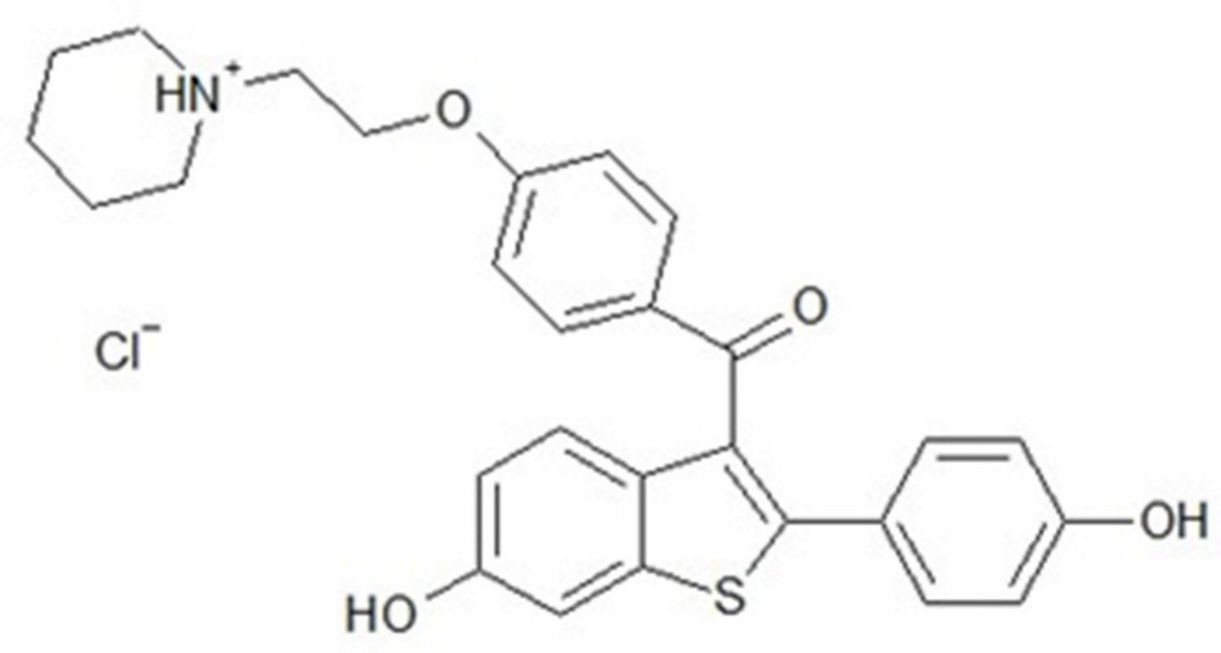
Chemical structure of RLX.

Raloxifene (RLX, Fig 1) is a selective estrogen receptor modulator and acts as a mixed estrogen agonist/antagonist [1–3]. RLX capabilities as an estrogen agonist in some tissues and as an estrogen antagonist in others (endometrium and breasts) generate several of estrogen’s beneficial consequences [3,4]. RLX is subjected to presystemic glucuronide conjugation after oral administration, which leads to reduced absolute bioavailability to 2% [5,6]. To avoid these drawbacks of oral RLX delivery, an alternative route of administration is considered. Transdermal drug delivery is considered a leading peroral alternative for its benefits in bypassing the presystemic metabolism of drugs, prolonging their effect, and reducing inter- and intrasubject variability. Ideal drug for transdermal delivery should meet some physicochemical properties for application in transdermal [7]. The physicochemical properties of RLX meets some criteria for the currently approved drugs for transdermal delivery with molecular weight of 473.6 g/mol, pKa (Strongest Acidic) of 8.89, pKa (Strongest Basic) of 7.95, Log p of 5.45, a dose of 60 mg PO/ Day with very low bioavailability of 2%. Previous reports have investigated transdermal and nasal delivery of RLX [5,8–11]. RLX was formulated as ethosomes transferosomes, and nanoparticles loaded transdermal films.

Transdermal drug conveyance, if successful, is a promising alternative for medications with low oral bioavailability and can also offer a more patient-convenient dosage form [12]. The skin is normally acting as a barrier to exceedingly hydrophilic or lipophilic materials. Over the past few decades, different techniques have been examined to overcome skin porousness obstruction[13,14]. Among the approaches used is the utilization of nanometer-sized transporters in transdermal medication delivery [15,16]. Nanocarriers used to improve transdermal conveyance are colloids, lipid-and polymer-based micellar arrangements, liposomes, transferosomes, ethosomes, cubosomes, solid lipid nanoparticles even with RLX nanostructured lipid transporter, and polymeric nanoparticles. [17,18]

Transferosomes are among the successful approaches for enhancing the drug flux through the skin through interaction with the lipid in stratum corneum and enhances the drug penetration. [19–21] The ultradeformable nature of transferosomes participates in the efficiency of transdermal delivery as a carrier system. The role of edge activator (surfactant) in these vesicles with the phospholipid participates in the elastic nature of these vesicles. D-α-tocopheryl polyethylene glycol 1000 succinate (TPGS) is a biocompatible and antioxidant surface active agent that has been approved by the FDA as a drug solubilizer in drug delivery systems.[19] TAT conjugating with phospholipids as liposomes was previously reported, but only few studies used TAT for translocation of drugs into cells via transdermal route. For example, TAT conjugated with polymeric lipid vesicles, which achieved high entrapment efficiency of hydrophilic therapeutic agents, and isolation of encapsulated drugs from the surrounding environment. In addition, higher stability and stronger ability to get through the stratum corium than using a formula without TAT. [22,23].

The aim of this work was the improvement of RLX bioavailability by enhancing its solubility and cellular penetration through formulation of TPGS-based transferosomes and augmenting its effect with the cationic cell-penetrating peptide CPP (TAT). Particle size, zeta potential, and transmission electron microscope investigation of the formed nanocarriers were measured. Further, drug release and cell viability studies were carried out.

## Materials and methods

### Materials

RLX and TPGS were purchased from Sigma-Aldrich (St. Louis, MI, USA). HIV-1 TAT protein was purchased from Chengdu Youngshe Chemical Co., Ltd. (Mainland, China). Phospholipon^®^ 90H (phospholipids) was gifted by Nattermann (Berlin, Germany).

### Formulation of RLX TPGS-transferosomes

RLX transferosomes were prepared as previously described with slight modification[19,24]. Phospholipon^®^ 90H (400 mg), 40 mg of TPGS, and 20 mg of RLX were dissolved in 30 mL ethanol. The alcoholic solution was removed under vacuum at 30°C. The film was agitated with 20 ml of deionized water containing 30 mg TAT and left for 3 h at ambient temperature to swell and then sonicated for 1 min using a probe sonicator to form the TPGS-based transferosomes-TAT. Plain TPGs-transferosomes and TPGs-transferosomes without TAT were prepared for comparative investigation.

### Transferosomes—Characterization

Size, size distribution (polydispersity index, PDI) and zeta potential of transferosomes were measured using Zetasizer (Malvern Instruments, Malvern, UK) based on the dynamic light scattering method. Briefly, the transferosomes recovered from centrifugation were sonicated in 10 mL of deionized water for 10 min to break up the aggregation and were stirred (500 rpm) at room temperature overnight. Subsequently, 0.5 mL aliquot was diluted and re-stirred for 3 h in 9.5 mL of deionized water for size determination in triplicate.

### Particle size and zeta potential analysis

The prepared TPGS-transferosomes, with and without TAT, loaded RLX were investigated for particle size and zeta potential by using Zetasizer Nano ZSP (Malvern Panalytical Ltd, Malvern, United Kingdom. Experiments were performed in triplicate.

### RLX-TPGS-transferosomes-TAT entrapment efficiency

RLX entrapment efficiency percent (EE%) was determined using the indirect method, as described previously[20]. Briefly, RLX-TPGS-transferosomes-TAT aqueous dispersion samples were centrifuged at 100,000 rpm for 45 min at 4°C. The supernatant was further filtered through a 0.1 μm membrane filter. RLX concentration in the filtrate was calculated for the unentrapped RLX using high-performance liquid chromatography (HPLC) assay method, as will be described later in the text. For all experiments, three independent experiments were performed in triplicate.

### Transmission electron microscope investigation

A transmission electron microscope (TEM; JEM-100S, Jeol Ltd., Tokyo, Japan) was used to investigate the prepared TPGS-transferosomes. The vesicles’ morphological features were assessed using this device, and sample preparation took place using the negative staining technique, which is commonly used in diagnostic microscopy. Direct dispersion of the samples was undertaken using bi-distilled water, after which a copper grid coated with collodion film was submerged into the solution more than once. Following staining with 2% (w/v) phosphotungestic acid solution and drying at room temperature, the sample preparation process was complete. Therefore, the TEM investigation was conducted at 70 kV (100CX, Jeol Ltd., Tokyo, Japan).

### Vesicle deformability %

Deformability of the prepared RLX-TPGS-transferosomes- and RLX-TPGS-transferosomes-TAT was determined, as previously reported, with modification [25,26]. Briefly, each formula was extruded through a Microsep^™^ device of 100K membrane molecular weight cutoff (10 nm pore size, PALL Life Sciences, Fribourg, Switzerland) with centrifugation at 20,000 x g. Vesicles’ size was measured before and after the centrifugation process and deformability % according to the following equation:

$$Deformability\;\%=\frac{Vesicle\;size\;before\;centrfiugation-Vesicle\;size\;after\;centrfiugation}{Vesicle\;size\;before\;centrifugation}\times 100$$

### Stability study of the formulation

Physical stability of the TPGS-transferosomes formula was evaluated for particle size measurement and zeta potential determination. Formulations were kept at room temperature (22°C ± 2°C, 60% RH ± 5% RH) for one month and at (4°C ± 2°C, 20% RH) for three months.

### RLX-transferosomes loaded transdermal film formulation

Hydroxypropyl methylcellulose (HPMC) polymer at a concentration of 1.5% w/v was used as film-forming polymer. The prepared TPGS-transferosomes were added to the polymeric solution. Propylene glycol (2% v/v) was used as plasticizer, and citral (2% v/v) was used as permeation enhancer; in addition, all were included in the polymeric solution. The mixture was stirred for 2 h (magnetic stirrer) and left in the refrigerator for 24 h; after which, the mixture was poured into a petri dish and maintained at 40°C in an oven until complete water evaporation. Then, the prepared films were kept for 24 h in a humid environment for the films to retain their moisture content. The prepared films were raw RLX film, RLX-loaded TPGS-transferosomes film, and RLX-loaded TPGS-transferosomes-TAT film. Each film was loaded with (1 mg/ cm^2^) of RLX per film area. The prepared films thickness was 0.253 ± 0.023 mm.

### RLX-loaded transdermal films *ex vivo* permeation study

The Research Ethics Committee, Faculty of Pharmacy, King Abdulaziz University, approved the study protocol, this experiment was done according to ethical approval no. (PH-188-40). Which ensures the care and use of animals, according to the EU Directive 2010/63/EU on the protection of animals used for scientific purposes and Guiding Principle in Care and Use of Animals (DHEW publication NIH 80–23). Full thickness skin samples of 3 × 3 cm area from the abdominal region of shaved Wistar rats were excised and freed from subcutaneous fats and examined using magnifier to assure skin integrity.[27] The prepared transdermal films (raw RLX, RLX-loaded TPGS-transferosomes, and RLX-loaded TPGS-transferosomes-TAT, equivalent to 50 μg/cm^2^) were investigated for diffusion behavior using a Franz diffusion automated system (Microette Plus; Hanson Research, Chatsworth, CA, USA). The prepared skin was mounted between the donor and receptor compartments of the diffusion cells with the dermal side in direct contact with the receptor medium. Transdermal films were placed between donor and acceptor chambers and allowed to be diffused through excised rat skin to phosphate-buffered saline (receptor medium, 7 mL, pH (7) stirred at 400 rpm, maintained at 32°C±0.5°C to correspond to normal human skin temperature [28,29], and contains 0.05% sodium dodecyl sulfate to maintain sink condition [30]. Aliquots (2 mL) were collected at 0.5, 1, 2, 4, 8, and 12 h.

The collected diffused samples were analyzed using Agilent HPLC (1200 series, Germany). The separation of RLX was carried out on a reversed phase hypersil ODS column and determined with UV detection at 284 nm. Mobile phase A was ammonium acetate solution 0.154% (pH 4.0), and mobile phase B was acetonitrile [31]. The calibration curve range was 10–1000 ng/ml. The minimum detectable concentration (LOD) of RLX was found to be 2 ng/mL, whereas the limit of quantification (LOQ) was 10 ng/mL.

### RLX formulation skin penetration visualization using fluorescence laser microscope

Visualization investigation was carried out using the same procedure utilized in the *ex vivo* permeation study, with some modifications. Rhodamine B (Rh) was incorporated in the TPGS-transferosomes as a fluorescent dye instead of RLX at concentration of 0.1% (w/v) (Ahmed & Rizg, 2018). Rh-TPGS-transferosomes with and without TAT films and raw Rh loaded films were prepared as previously described in the transdermal film formulation part. Skin sections were taken out from the cells, after 1 and 4 h removed, immersed in and then kept in formaldehyde-phosphate-buffered saline (pH 7.4) 10% (v/v) for 24 h. Blocks of skin sample paraffin wax, 4 nm thick, sections were prepared using a microtome. Visualization was performed by a Zeiss Axio Observer D1 Inverted Dic fluorescence microscope (Carl Zeiss AG, Oberkochen, Germany). The filter used featured 470/40 nm excitation, 495 beam splitter, and 525/50 nm emission. Images were acquired with identical acquisition parameters, with minimum excitation and gain [32].

### RLX-TPGS-transferosomes-TAT Cytotoxicity against MCF-7 cells

MCF-7 breast cancer cells were gifted from Dr. Serag El-bahiry’s cell culture lab, Faculty of Science, King Khalid University, Abha, KSA, that was approved by the Research Ethics Committee, Faculty of Pharmacy, King Abdulaziz University. MCF-7 cells were grown in media composed of DMEM/Ham’s F12 supplemented with 5% fetal bovine serum (FBS), 2 mM L-glutamine, 100 μg/ml streptomycin, 100 μ/ml penicillin, and 2.2 g/l NaHCO3. For all procedures, cells were maintained at 37˚C in a humidified atmosphere of 5% CO2, as described by the manufacturer (Lonza, Auckland, New Zealand). Exponentially growing cells at a cell density of 5×104 cells/ml were tripsinized by 0.25% Trypsin-EDTA and seeded in 96 well plates at 2000–5000 cells/well. Cells were incubated for 24 h at 37°C in 5% CO2 and then treated with 0–30 μM of raloxifene or RL91. DMSO (0.1%) served as the vehicle control. Cells were incubated for 72 h and then fixed using trichloroacetic acid (TCA) 10% solution. Cell number was determined using the sulforhodamine B (SRB) assay[33]. The concentration required us to decrease cell number by 50% (IC50), as determined. Cells were treated with RLX, RLX-TPGS-transferosomes-TAT, and plain TPGS-transferosomes-TAT formulations in concentrations from (0.01–100 μg/ml). The experiments were carried out in triplicate, and results were expressed as cell number.

### Statistical analysis

The results will be presented as mean ± SD calculated over at least three data points. Statistical significance will be processed by one-way analysis of variance at *P* ≤ 0.05. Data will be analyzed using SPSS program version 16 (SPSS an IBM company, Chicago, IL, USA).

## Results and discussion

According to Table 1, the prepared transferosomes showed average particle size, measured by Zetasizer Nano ZSP of 33.1, 36.9, and 96.05 nm for plain TPGS-transferosomes, RLX-TPGS-transferosomes, and RLX-TPGS-transferosomes-TAT, respectively. Size distribution revealed an acceptable polydispersity index range for the prepared formulae (Table 1). Zeta potential results revealed positively charged vesicles of 39.4 mV for TPGS-transferosomes-TAT (Table 1). In addition, results of RLX EE % showed no significant change in the EE % for both formulations with 89.34± 2.67% and 90.18 ± 4.57% for TPGS-transferosomes and TPGS-transferosomes-TAT, respectively (Table 1).

**Table 1:** Size, zeta potential, PDI and EE % of TPGS-transferosomes (Plain), RLX-TPGS-transferosomes and RLX-TPGS-transferosomes-TAT formulations. Results represents Mean ±SD.

Formula	Size (nm)	Zeta potential (mV)	PDI	EE %
**TPGS-transferosomes (Plain)**	33.1±2.1	2.3±0.2	0.37±0.02	----------
**RLX-TPGS-transferosomes**	36.9±3.4	8.1±1.1	0.38±0.04	89.34± 2.7%
**RLX-TPGS-transferosomes-TAT**	96.05±4.3	39.4±2.3	0.49±0.06	90.18 ± 4.6%

**Fig 2:**
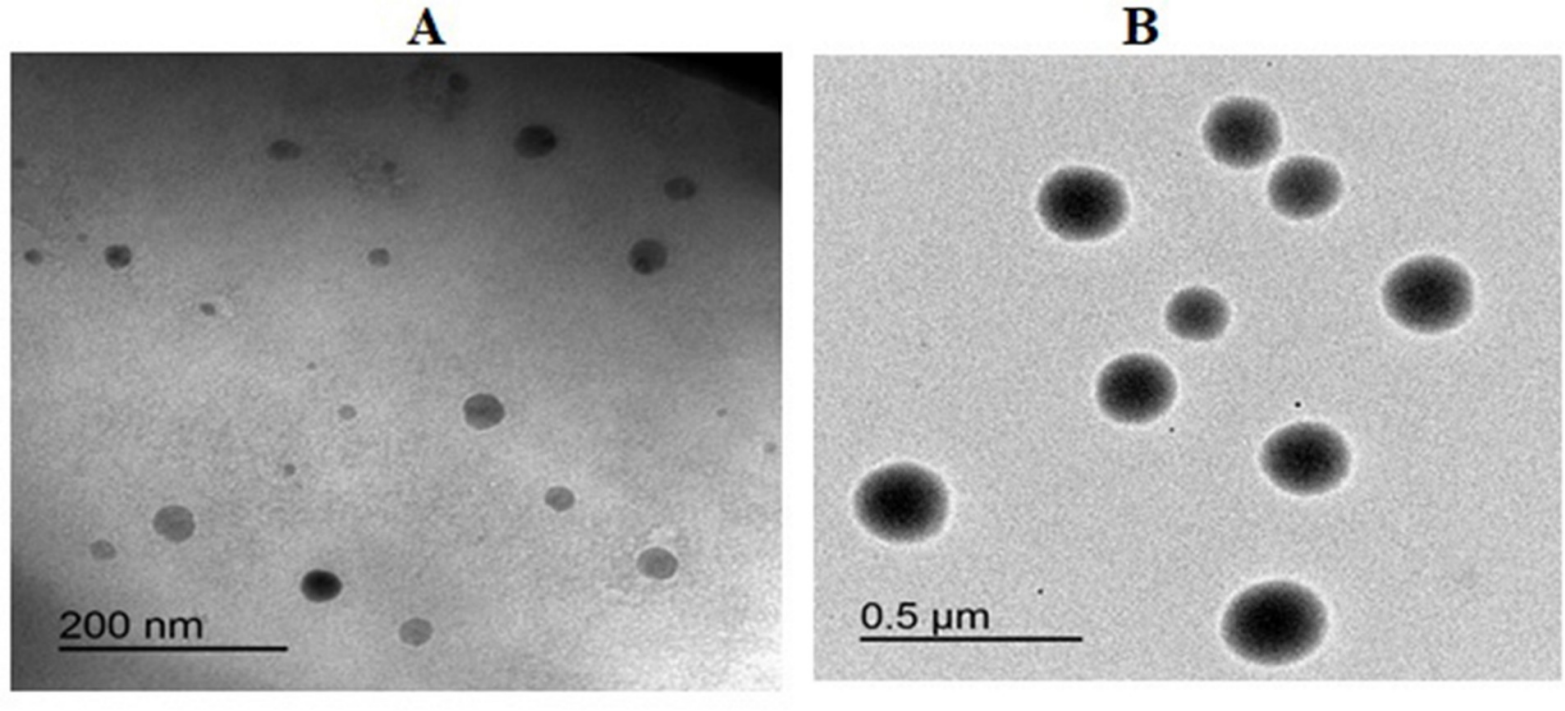
TEM images of RLX-TPGS-transferosomes (A) and RLX-TPGS-transferosomes-TAT (B).

**Fig 3:**
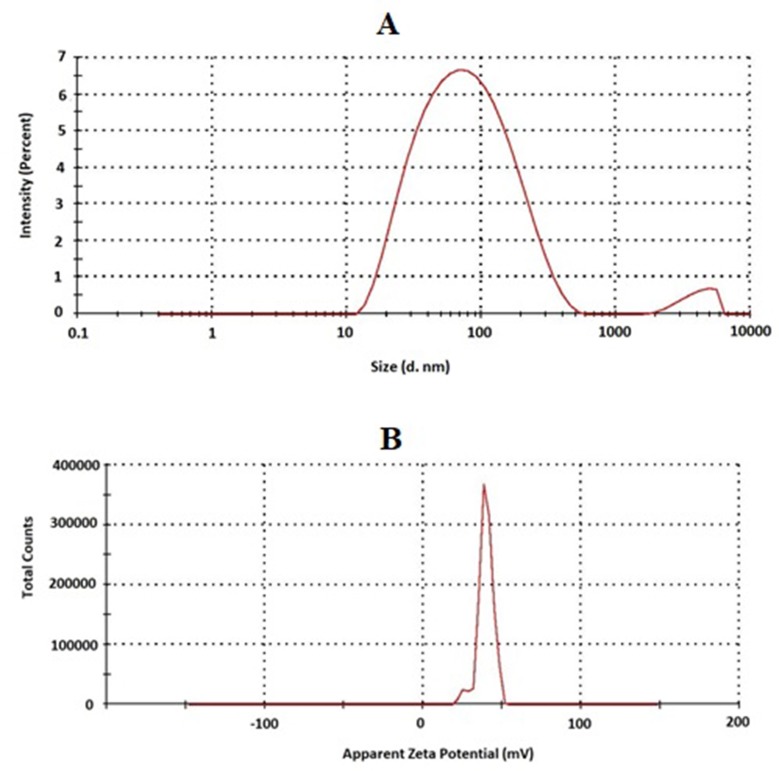
Size (A) and zeta potential (B) distribution of RLX-TPGS-transferosomes -TAT.

RLX transferosomes TEM images (Fig 2) and also, size and zeta distribution for TPGS-transferosomes-TAT (Fig 3) revealed spherical vesicles with comparable average vesicle. Vesicles deformability % indicated that the % change results revealed 15.47% and 21.14% for RLX-TPGS-transferosomes and RLX-TPGS-transferosomes-TAT, respectively. Stability studies of the prepared RLX transferosomes revealed no significant change (*p* < 0.05) in vesicles’ size and zeta potential after 1 and 3 months. Results revealed no significant difference (*p* < 0.05) between plain TPGS-transferosomes and RLX-TPGS-transferosomes in particle size. On the other hand, RLX-TPGS-transferosomes-TAT showed significant increase in vesicular size when compared with the other investigated formulae. The marked increase in zeta potential, positive value, of the prepared RLX-TPGS-transferosomes-TAT is attributed to the inclusion of the positively charged TAT protein. TAT proteins are a group of proteins characterized by arginine-rich motif (ARM) RNA binding proteins [34]. The improved zeta potential of RLX-TPGS-transferosomes-TAT (39.4 mV) will enhance cellular uptake of the prepared vesicles and improved delivery of RLX[1–4,35]. Improved EE% of RLX-transferosomes formulations could be attributed to the low RLX water solubility that favors its entrapment within the vesicles. The slight change in deformability percentage could be attribute to the inclusion of TAT to the RLX-TPGS-transferosomes-TAT vesicles formula that could have imparted relative rigidity when compared with RLX-TPGS-transferosomes. Stability studies for the prepared RLX transferosomes indicated stabilization of the prepared nano-dispersion upon storage.

**Fig 4:**
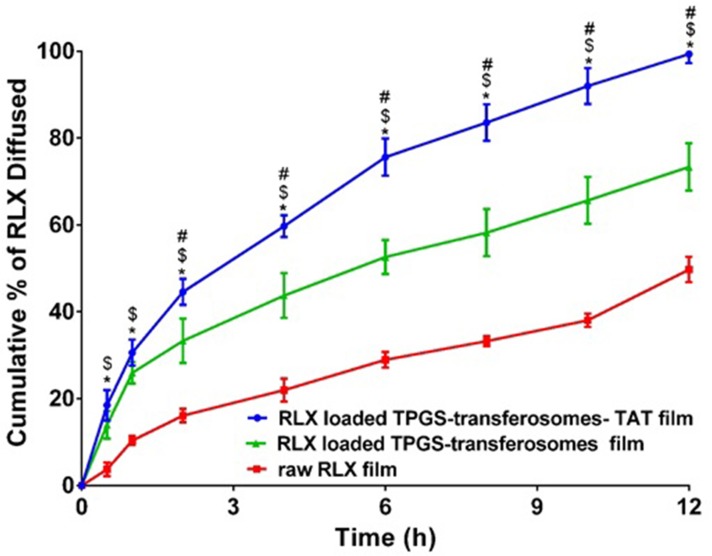
Permeation of raw RLX transdermal film, RLX-TPGS-transferosomes film and RLX-TPGS-transferosomes-TAT film through rat skin. **Notes:** Value indicate the mean ± SD (n = 3). # p < 0.05 RLX-TPGS-transferosomes-TAT compared to RLX-TPGS-transferosomes formula; * p < 0.05 RLX-TPGS-transferosomes-TAT compared to raw RLX; $ p < 0.05 RLX-TPGS-transferosomes formula compared to raw RLX.

TPGS-transferosomes have the advantage of improving the solubility of RLX as a result of the solubilizing ability of TPGS [5]. Also, the antioxidant activity of TPGS could protect the oxidative degradation of RLX during storage [36,37]. In addition, TPGS is utilized as a P-glycoprotein (P-gp) inhibitor to overcome multidrug resistance and for improving oral drug formulations bioavailability [38]. It is also previously reported to be expressed in the skin that could be utilized in improving transdermal drug delivery [39]. The ultradeformable nature of transferosomes, as a result edge activator, enhances squeezing and penetration of vesicular structure through the skin [16,40]. In addition, the positively charged TAT characters of TPGS-transferosomes-TAT improves RLX delivery across skin layers. The inclusion of TAT to the transferosomal formulation increases the average vesicular size compared with RLX-TPGS-transferosomes formula, as shown in Fig 4, which could be attributed to the inclusion of TAT on the surface of the prepared vesicles, which leads to increase in the bulk volume of the vesicles. The diffusion of RLX-TPGS-transferosomes-TAT loaded film was compared with RLX-TPGS-transferosomes loaded film and raw RLX loaded film. The permeation was investigated through rat skin. The results revealed significant (*p* < 0.05) enhancement of RLX permeation through rat skin from RLX-TPGS-transferosomes-TAT loaded film when compared with RLX-TPGS-transferosomes loaded film and raw RLX film Fig 4. The results revealed permeation of RLX after 2 h of 44.63%, 33.33%, and 16.12% from RLX-TPGS-transferosomes-TAT loaded film, RLX-TPGS-transferosomes loaded film and raw RLX loaded film, respectively. RLX-TPGS-transferosomes-TAT loaded film showed permeation of 99.33% after 12 h when compared with 49.73% for raw RLX loaded film.

**Fig 5:**
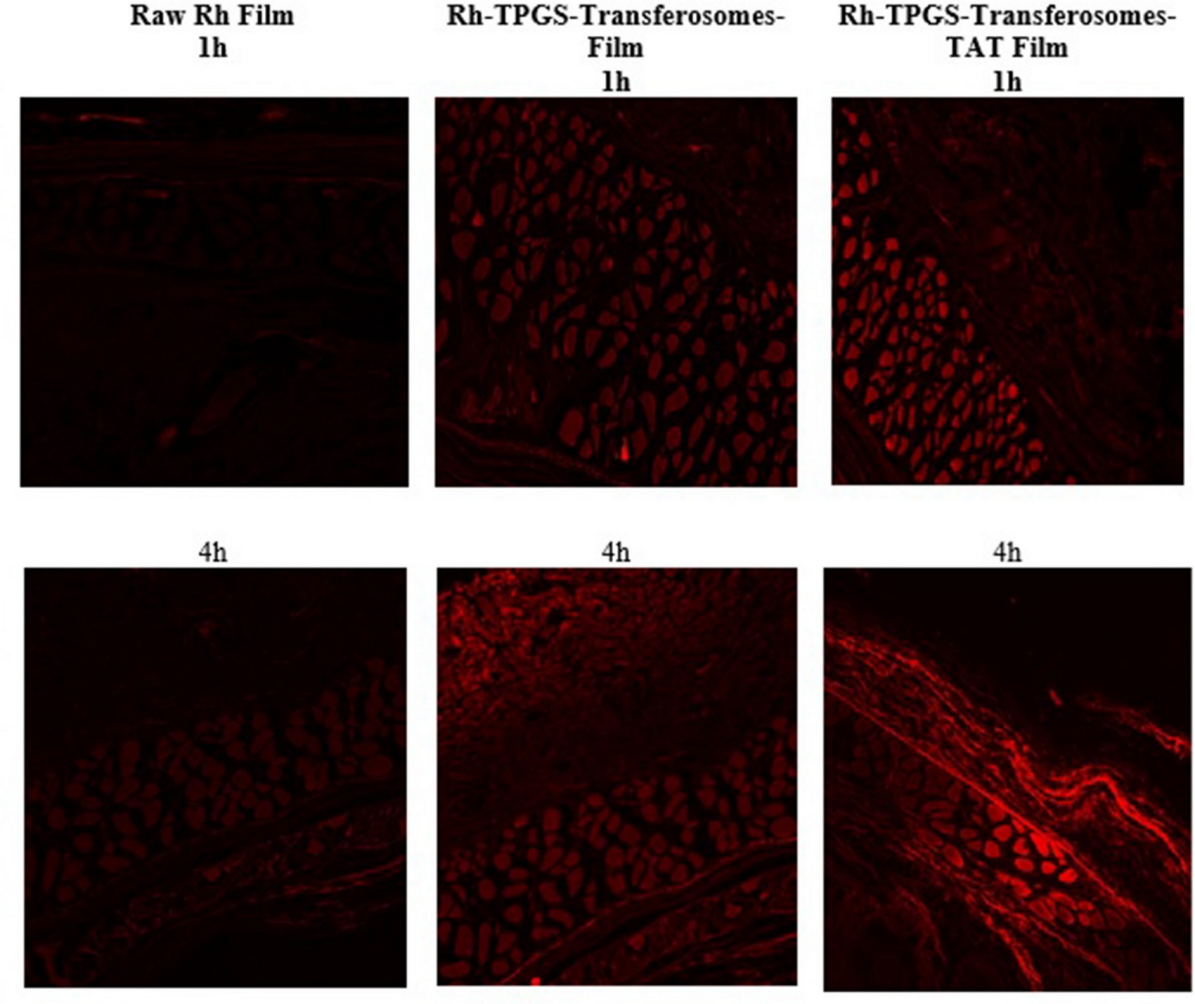
Fluorescence laser microscopy images for the permeation of raw Rh transdermal film (left column), and Rh-TPGS-Transferosomes Film (middle column) and Rh-TPGS-Transferosomes-TAT Film (right column) through rat skin after 1 and 4 hours. Magnification 400×.

Fluorescence laser microscope images of Rh-TPGS-transferosomes-TAT film showed enhanced skin layers penetration over raw Rh film. The results showed widespread fluorescence intensity distribution through skin layers after 1 h and 4 h for transferosomes formula, as shown in Fig 5. The fluorescence laser microscope results (Fig 5) showed the penetration enhancement of Rh-TPGS-transferosomes-TAT transdermal film through different layers of rat skin. The marked permeation enhancement is attributed to the characters (ability) of the transferosomes formula to penetrate skin layers as a result of its elastic and ultradeformable nature of the nanostructured formula when compared with raw transdermal film[24,41,42]. TPGS as edge activator improves transferosomal structural flexibility that improves vesicular squeezing and penetration through the skin layers [20].

**Fig 6:**
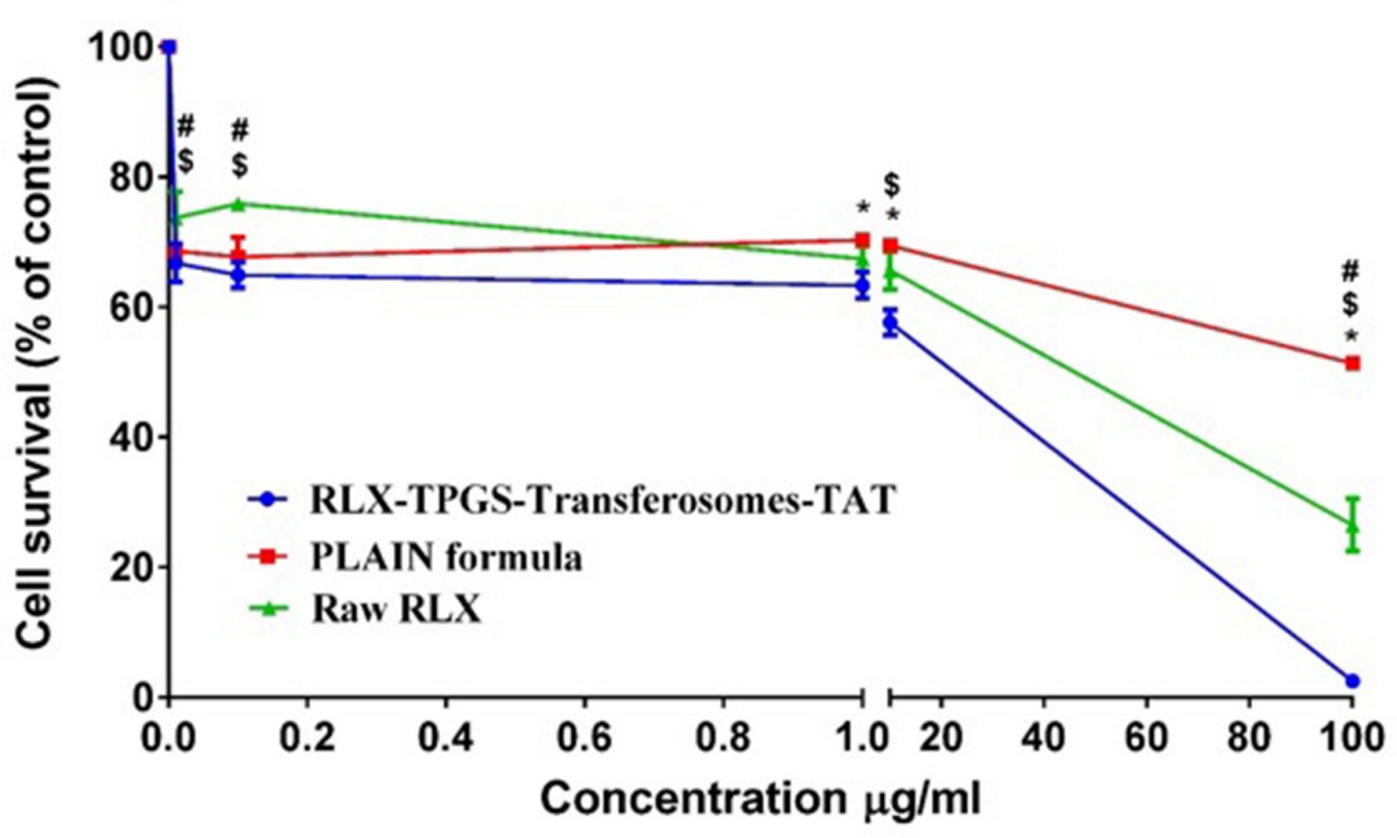
IC_50_% of RLX-TPGS-transferosomes-TAT, plain TPGS-transferosomes-TAT formula and raw RLX. Notes: Value indicate the mean ± SD (n = 6). * p < 0.05 RLX-TPGS-transferosomes-TAT compared to plain TPGS-transferosomes-TAT formula; $ p < 0.05 RLX-TPGS-transferosomes-TAT compared to raw RLX; # p < 0.05 plain TPGS-transferosomes-TAT formula compared to raw RLX.

To provide the potency of RLX-TPGS-transferosomes-TAT and raw RLX and plain formula in the MCF-7 cell lines, IC50 curves were initially produced. Both raw RLX and RLX-TPGS-transferosomes-TAT showed consistent cytotoxicity across the cell lines, but RLX-TPGS-transferosomes-TAT was more potent than raw RLX. Specifically, IC50 values for raw RLX was 2.77 ± 0.38 μM, while the raw RLX—TPGS-transferosomes-TAT was 1.98 ± 0.17 μM, as shown in Fig 6. TAT increases inclusion of RLX transferosomes and improves cellular RLX uptake via attraction with the negatively charged cell membrane. It is possible to use the improved cellular cytotoxicity and penetration of RLX, facilitated due to TAT, to promote the effectiveness of the standard RLX dose in MCF-7 cancer cells and, additionally, to mitigate the secondary effects of RLX.

The uptake of human cells occurs with TAT peptides through a range of pathways, which can emerge at the same time. TAT, given its status as a molecule with a positive charge, heightens the inclusion of RLX transferosomes and, moreover, enhances cellular RLX uptake based on its attraction to the cell membrane, which has a negative charge. This results in the entrance of the cellular cytoplasm via the receptor-independent pathway. More specifically, the link between the cellular membrane and TAT incorporates the attraction between proteoglycans, which have a negative charge, and simple amino acids, which have a positive charge. Furthermore, the stimulation of intracellular signalling cascades takes place due to TAT, which results in the improvement of the uptake process’s organic pathway. As an energy-structured and natural process, endocytosis can start with interactions between negative and positive charges. This is allocated in proteoglycans on the cell surface, as well as the TAT peptide, which has an effect on the degree to which the lipid bilayer is stable. Additionally, the ability of TAT to enter cells based on unique endocytic pathways, particularly pinocytosis, is high. The selection of an endocytic uptake mechanism is dependent on factors such as the load features of the transferosome, the characteristics of the peptide, and the goal characters that are distinctive to the cell [35, 37, 38]. The confining of RLX transport occurs due to the help of the metabolism of the extracellular carrier system, and the uptake of peptides is implicated in this process [39]. Avoiding lysosome degradation relies on endosomal escape, since this allows it to arrive at the required biological site. These findings are expected to enhance the ability of future studies to examine the part played by cationic peptide in the augmentation of transdermal delivery. Furthermore, novel approaches to RLX delivery routes can be pursued to increase the degree to which drugs are bioavailable and effective.

## Conclusion

The study confirms the synergistic effect of TAT in transferosome form on RLX cytotoxicity against MCF-7 breast cancer cells. Moreover, particle size has an impact on cellular cytotoxicity. The prepared transferosome achieved a relatively high EE% with a relatively release profile. Cell viability confirmed that the incidence of cytotoxicity augmentation by about 42%. Finally, loading RLX and TAT on TPGS transferosomes may provide a novel strategy for RLX delivery to prevent the occurrence of breast cancer.
