# Expression of Concern: Vitamin E TPGS based transferosomes augmented TAT as a promising delivery system for improved transdermal delivery of raloxifene

**DOI:** 10.1371/journal.pone.0291080

**Published:** 2023-08-30

**Authors:** The PLOS ONE Editors

Following the publication of this article [1], concerns were raised regarding results presented in Figs 2 and 5. Specifically,

‐ The Fig 2B panel in this article appears to overlap with Figure 2 of [2] and the Figure 1B panel of [3].
‐ The Fig 5 Raw Rh film 4h panel appears to partially overlap with the Fig 5 Rh-TPGS-Transferosomes-Film 4h panel.

The authors commented that the TEM images in this article are correct, and that they were inadvertently duplicated in [2, 3]. Furthermore, the authors stated that the incorrect panel was used for the Fig 5 Raw Rh film 4h results and provided an updated Fig 5 with the correct image, which is included with this notice. The raw data underlying the Figure 2, 4, 5, and 6 results are provided in the S1–S4 Files.

**Fig 5:**
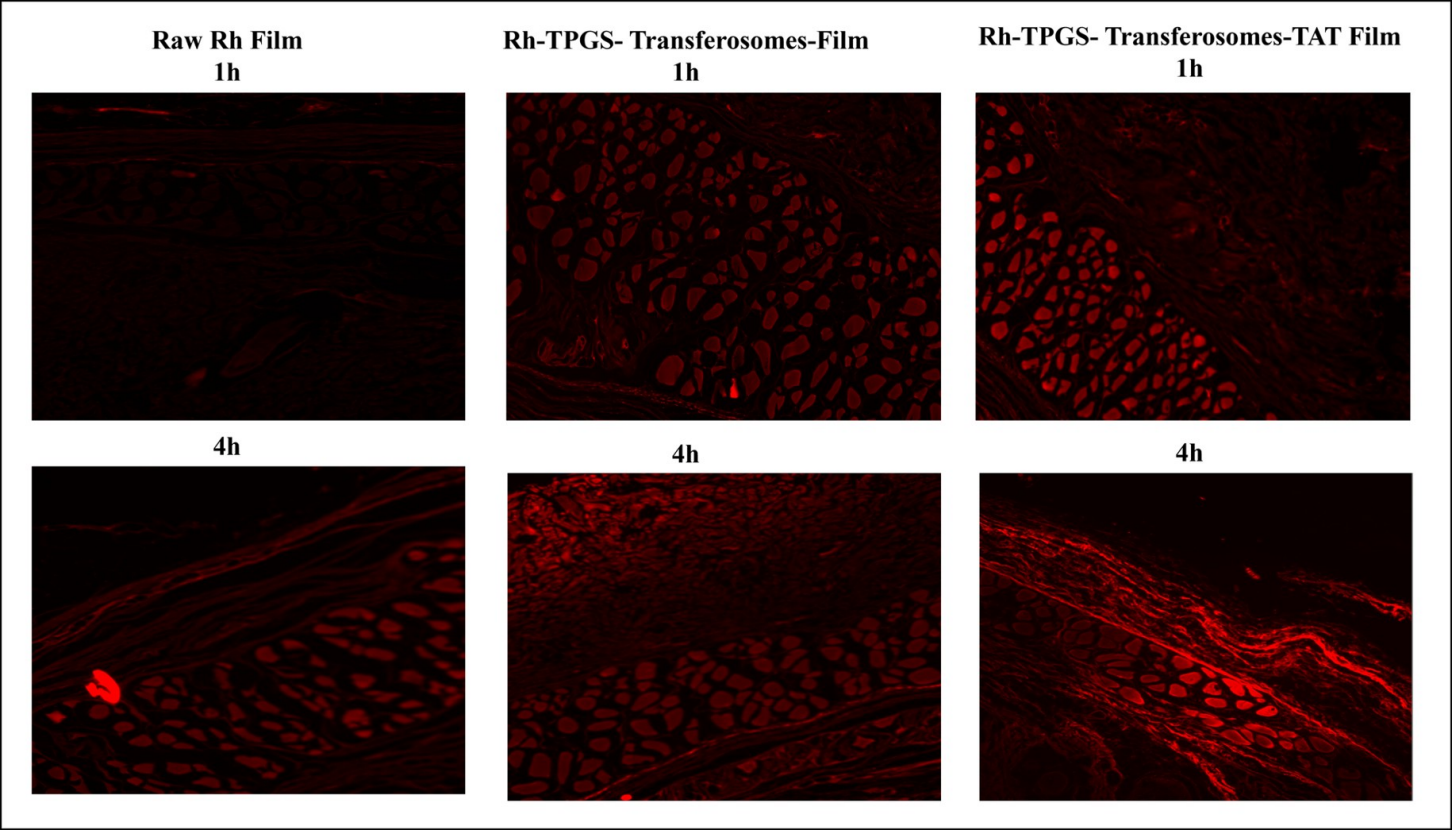
Fluorescence laser microscopy images for the permeation of raw Rh transdermal film (left column), and Rh-TPGS-Transferosomes Film (middle column) and Rh-TPGS-Transferosomes-TAT Film (right column) through rat skin after 1 and 4 hours. Magnification 400×.

The corresponding author stated that during the data collection for the Fig 5 results, multiple images were captured of the same samples so that the highest resolution images could be used for publication. However, due to a coding error of the data files, some images unintentionally overlapped.

Editorial assessment of the Fig 5 underlying data provided by the authors highlighted additional panel overlap concerns, as one of the raw underlying images provided for the Rh-TPGS Transferosomes 4h results appears to partially overlap with one of the raw underlying images provided for the Rh-TPGS Transferosomes TAT 1h results. The authors stated that the additional image overlap in the underlying data is also the result of the unintentional coding error. In light of these coding errors, the reliability of the Fig 5 results is in question, and these data should be interpreted with caution.

The quantitative data underlying other results reporting in the article are available upon request from the corresponding author.

In light of the concerns raised both with the article and the underlying data provided, the *PLOS ONE* Editors issue this Expression of Concern to notify readers of the above concerns and to relay the available data provided by the corresponding author.

## Supporting information

S1 File Original data underlying Fig 2. (ZIP)

S2 File Original data underlying Fig 4. (ZIP)

S3 File Original data underlying Fig 5. (ZIP)

S4 File Original data underlying Fig 6. (ZIP)
