# Supplementary figures and images for: Expression of Concern: Vitamin E TPGS based transferosomes augmented TAT as a promising delivery system for improved transdermal delivery of raloxifene
Source: PLoS One. 2023 Aug 30;18(8):e0291080. doi: 10.1371/journal.pone.0291080 (PMC10468055; doi:10.1371/journal.pone.0291080)

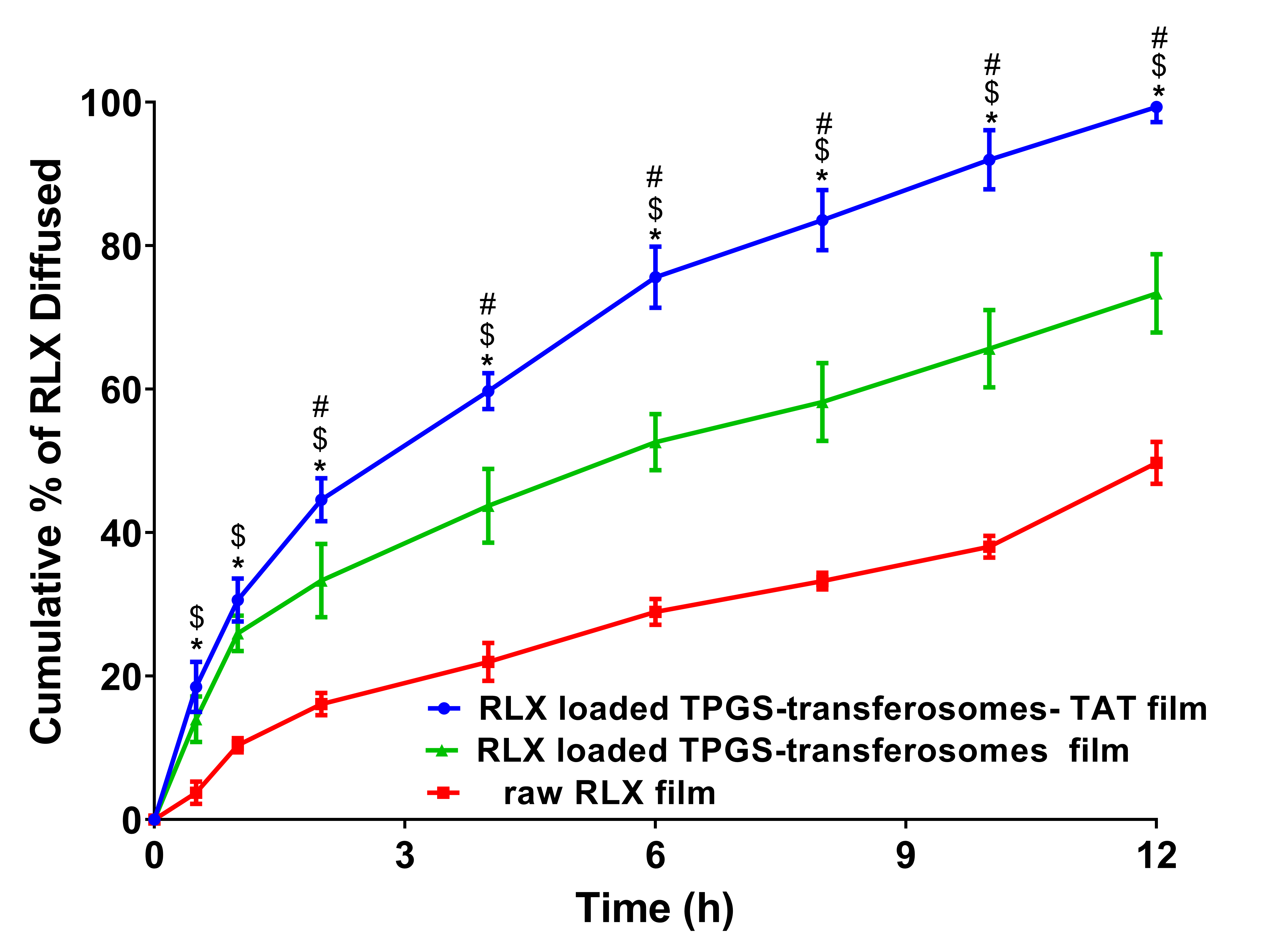

Supplement: S2 File — (ZIP) [file pone.0291080.s002.zip › Figure 4/Figure 4.tif]

## Slide 1
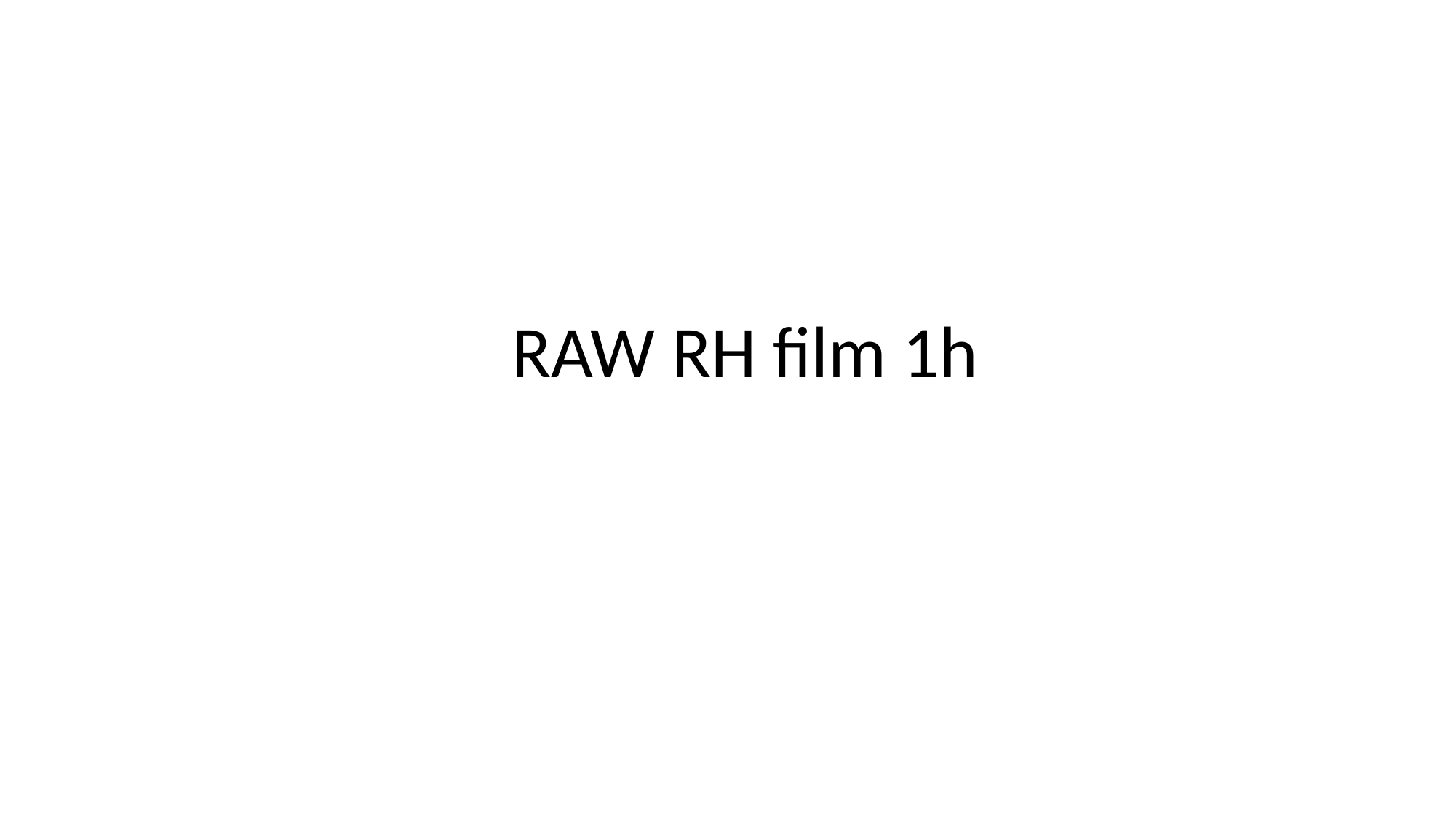

RAW RH film 1h

## Slide 2
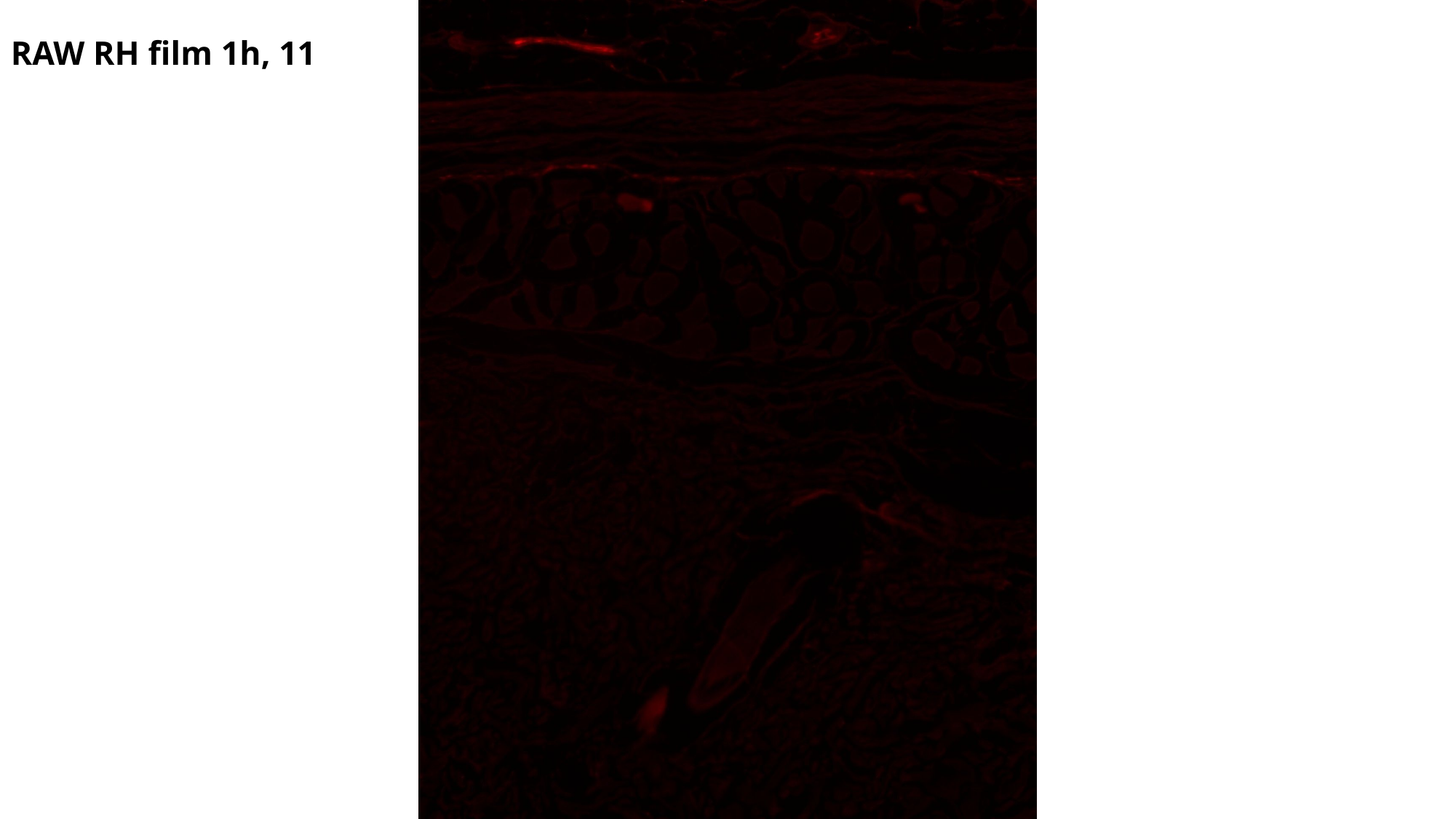

RAW RH film 1h, 11

## Slide 3
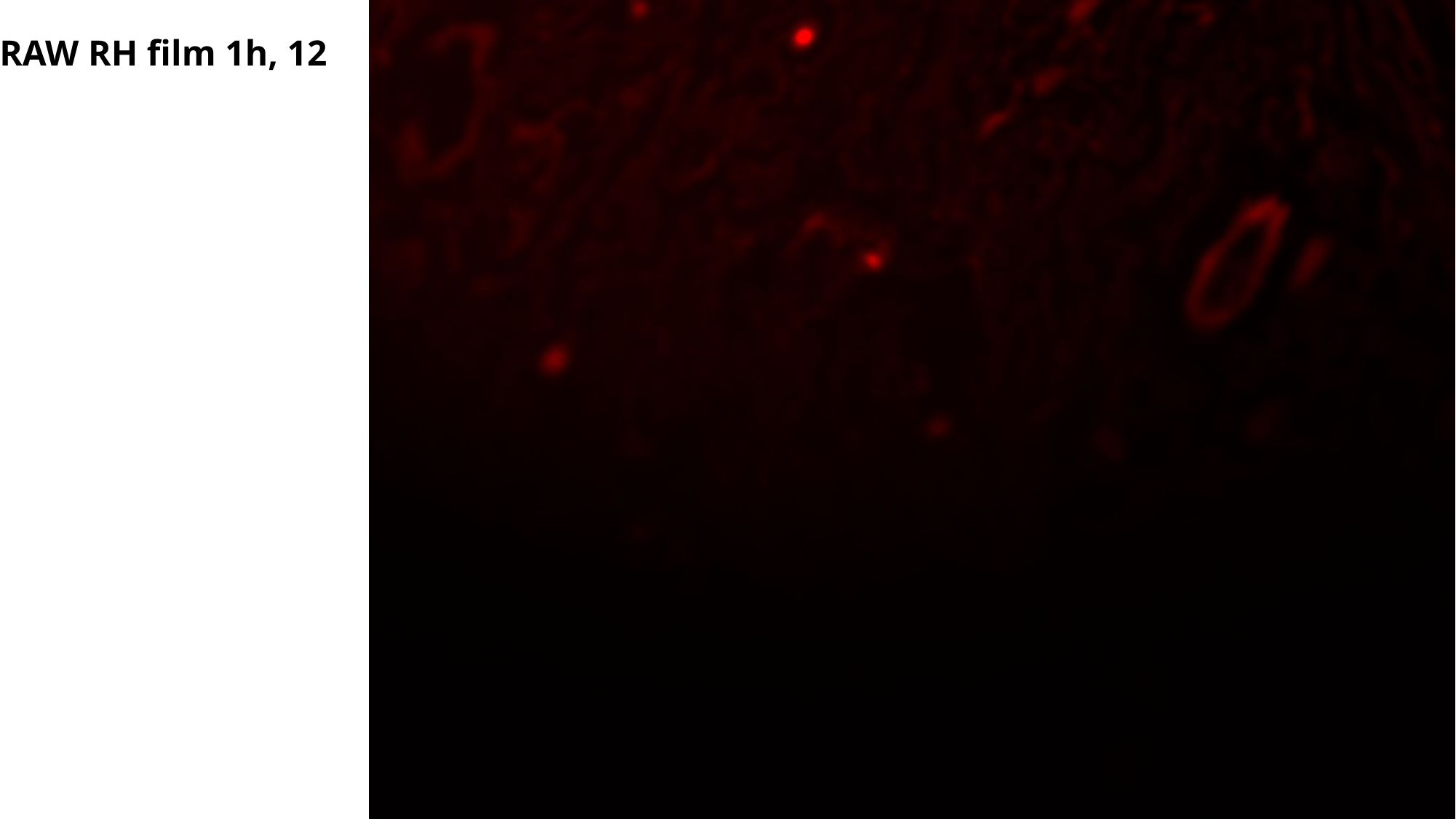

RAW RH film 1h, 12

Supplement: S3 File — (ZIP) [file pone.0291080.s003.zip › FIGURE 5 & Raw data/Figure 5 ALL Images from instrument/ALL RAW RH film 1h.pptx]

## Slide 1
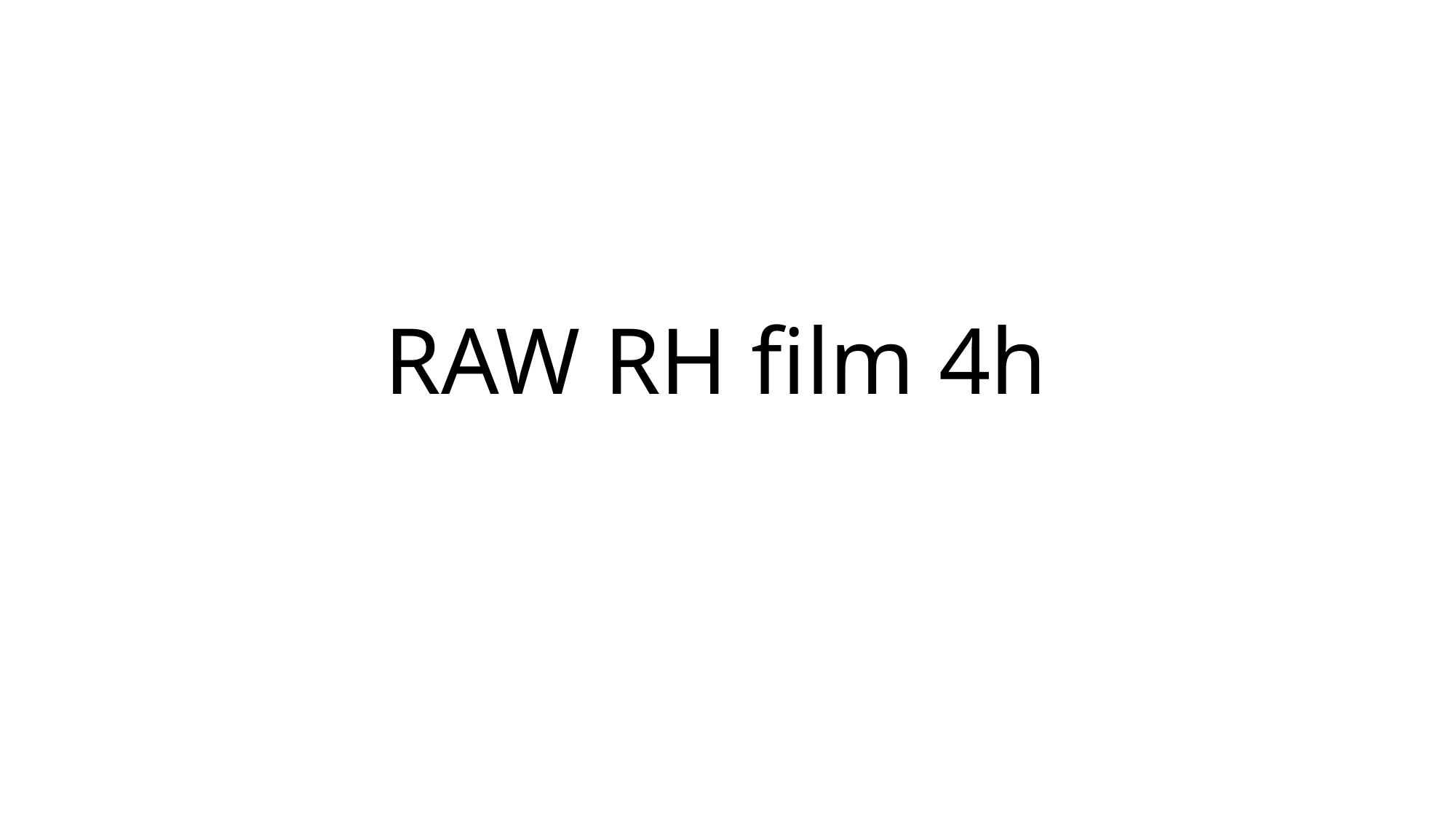

# RAW RH film 4h

## Slide 2
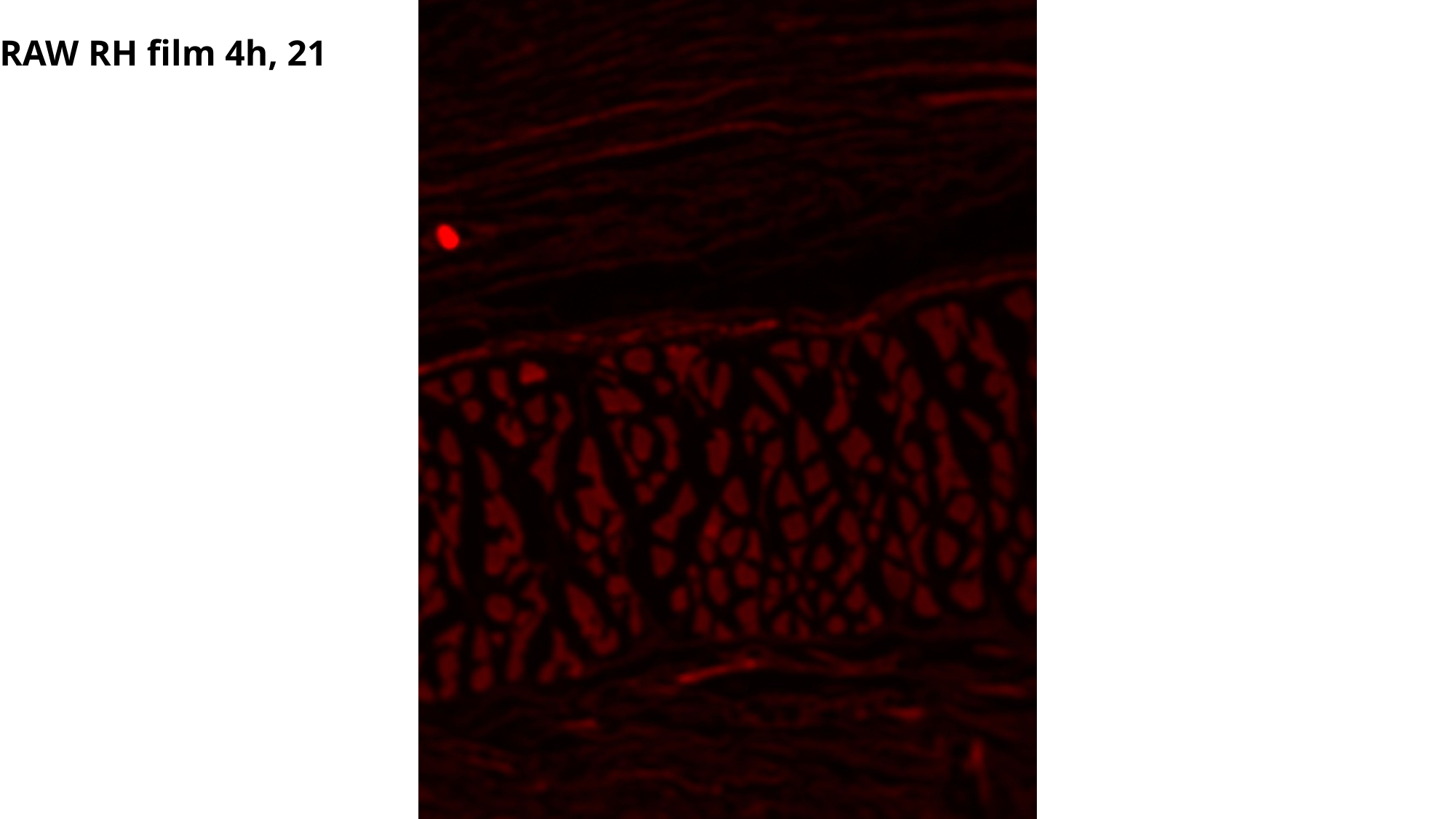

RAW RH film 4h, 21

## Slide 3
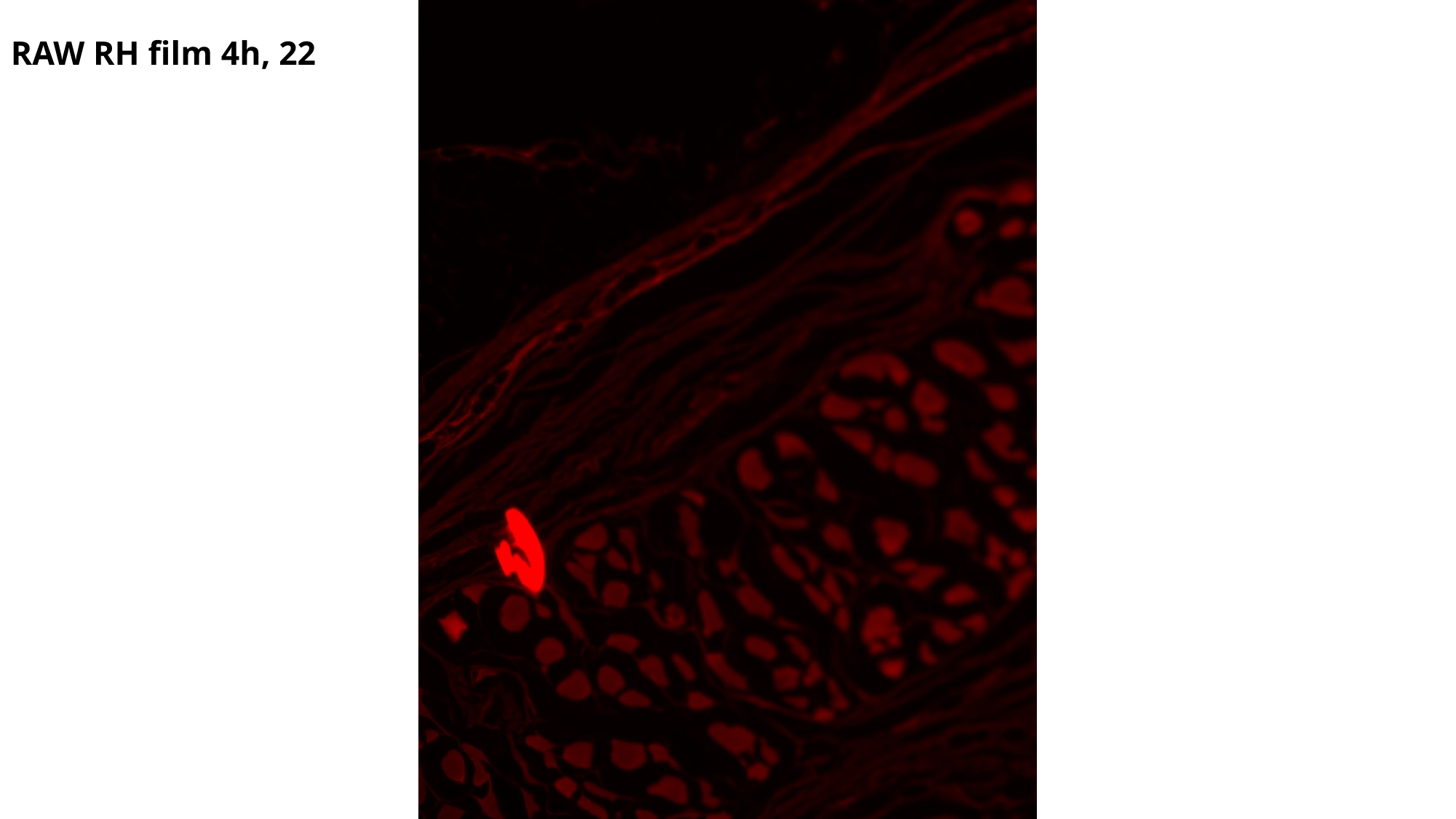

RAW RH film 4h, 22

## Slide 4
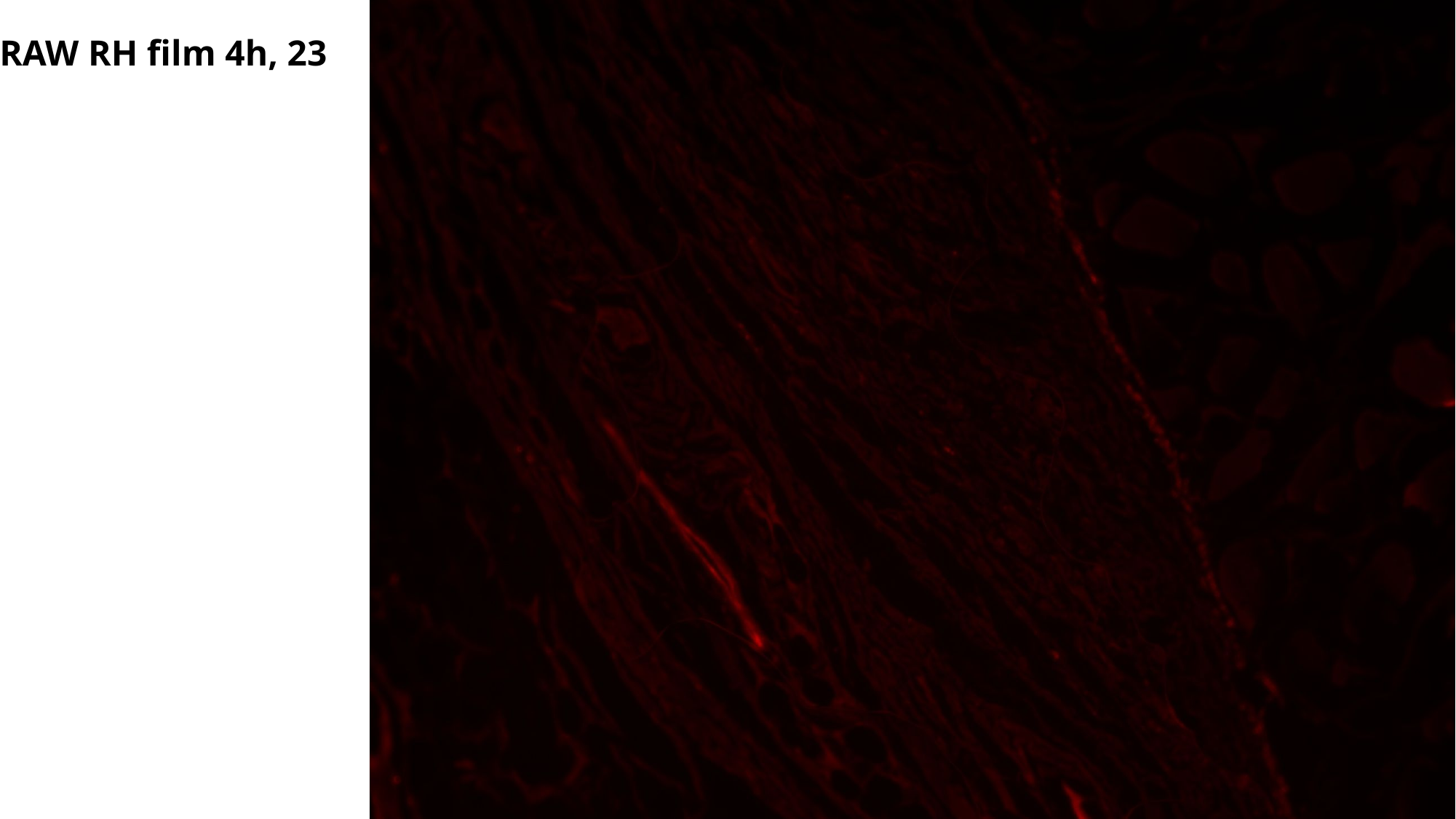

RAW RH film 4h, 23

Supplement: S3 File — (ZIP) [file pone.0291080.s003.zip › FIGURE 5 & Raw data/Figure 5 ALL Images from instrument/ALL RAW RH film 4h.pptx]

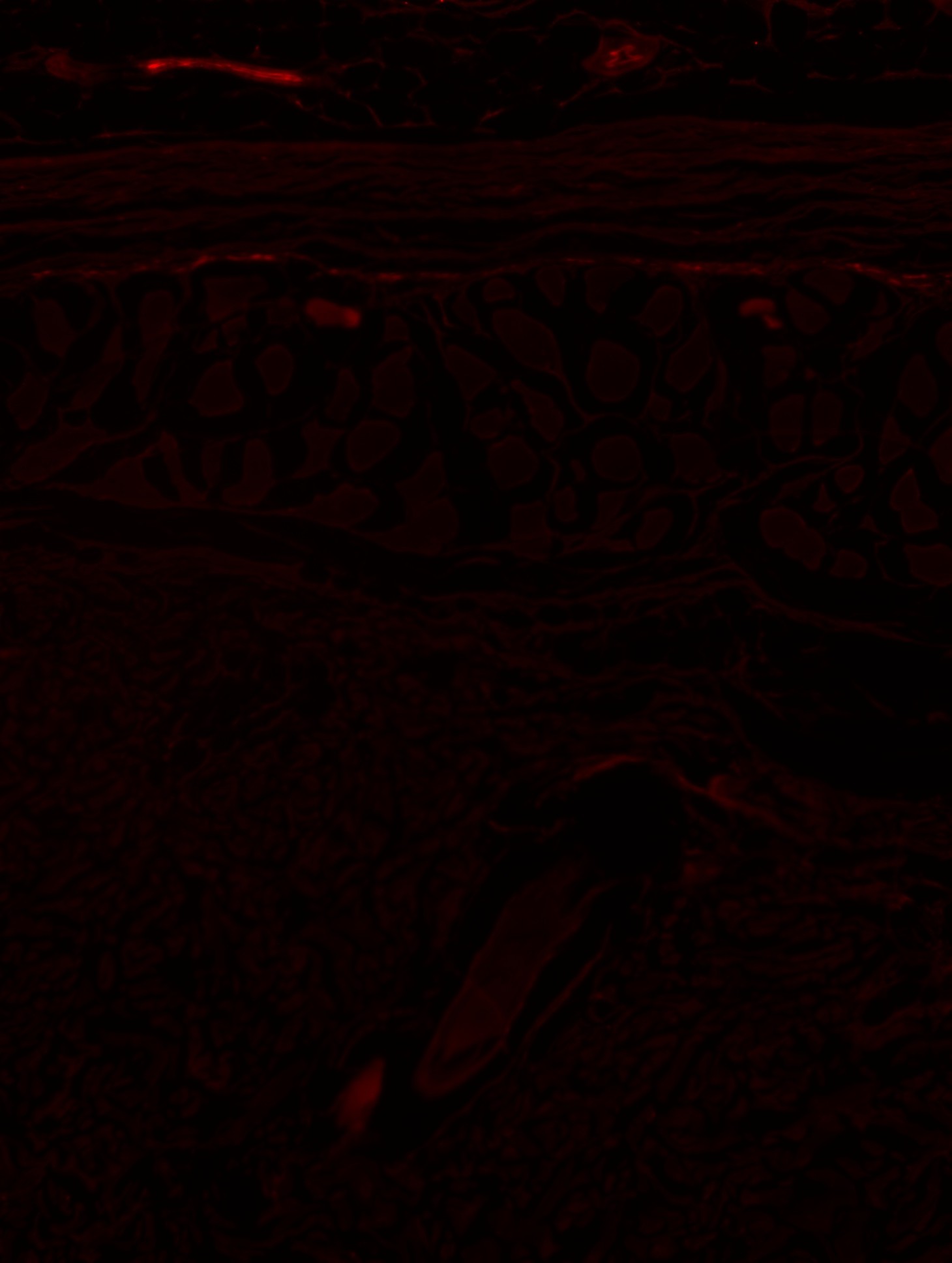

Supplement: S3 File — (ZIP) [file pone.0291080.s003.zip › FIGURE 5 & Raw data/Individual Figure 5 images/RAW RH film 1h.jpg]

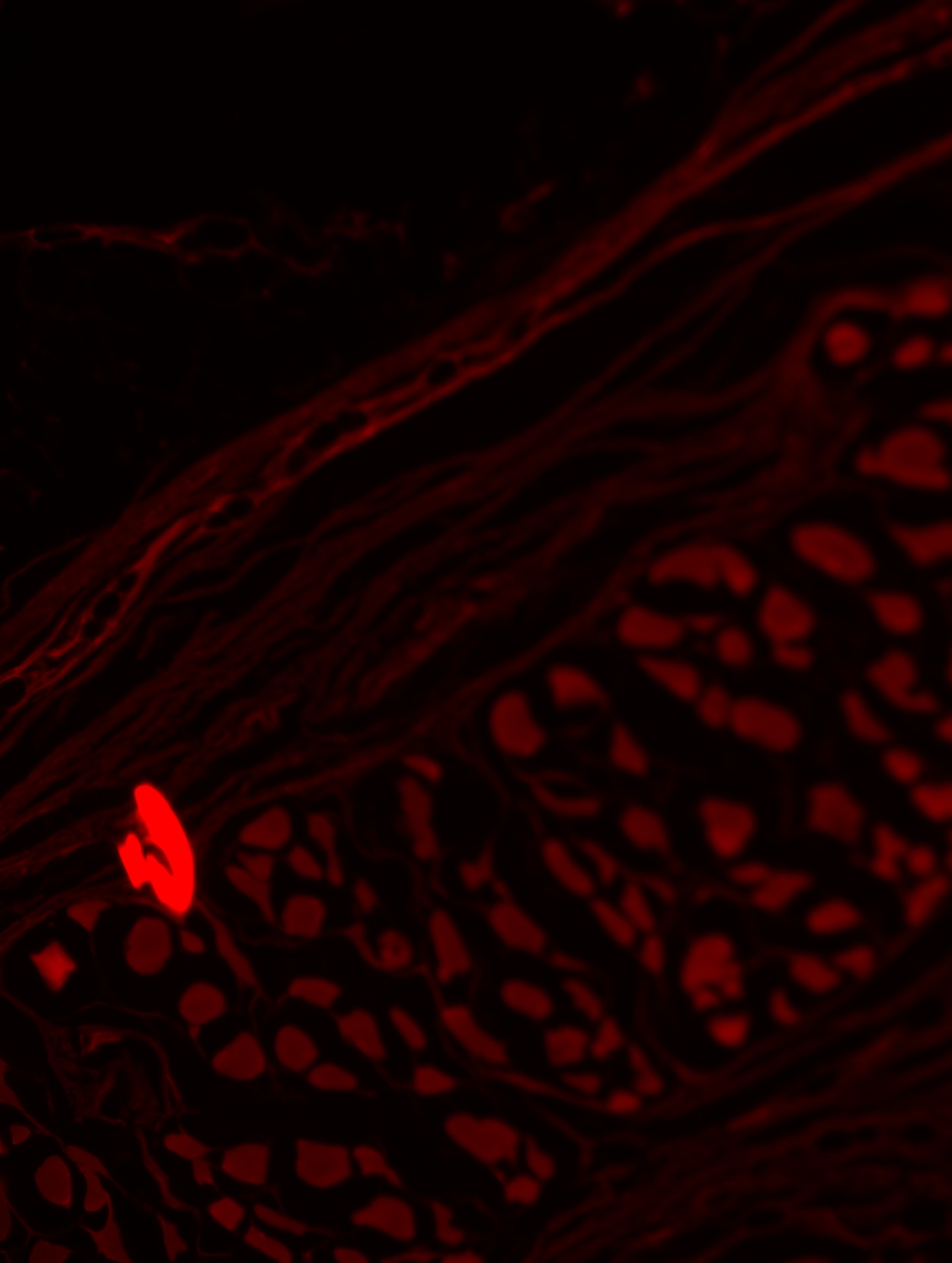

Supplement: S3 File — (ZIP) [file pone.0291080.s003.zip › FIGURE 5 & Raw data/Individual Figure 5 images/RAW RH film 4h.jpg]

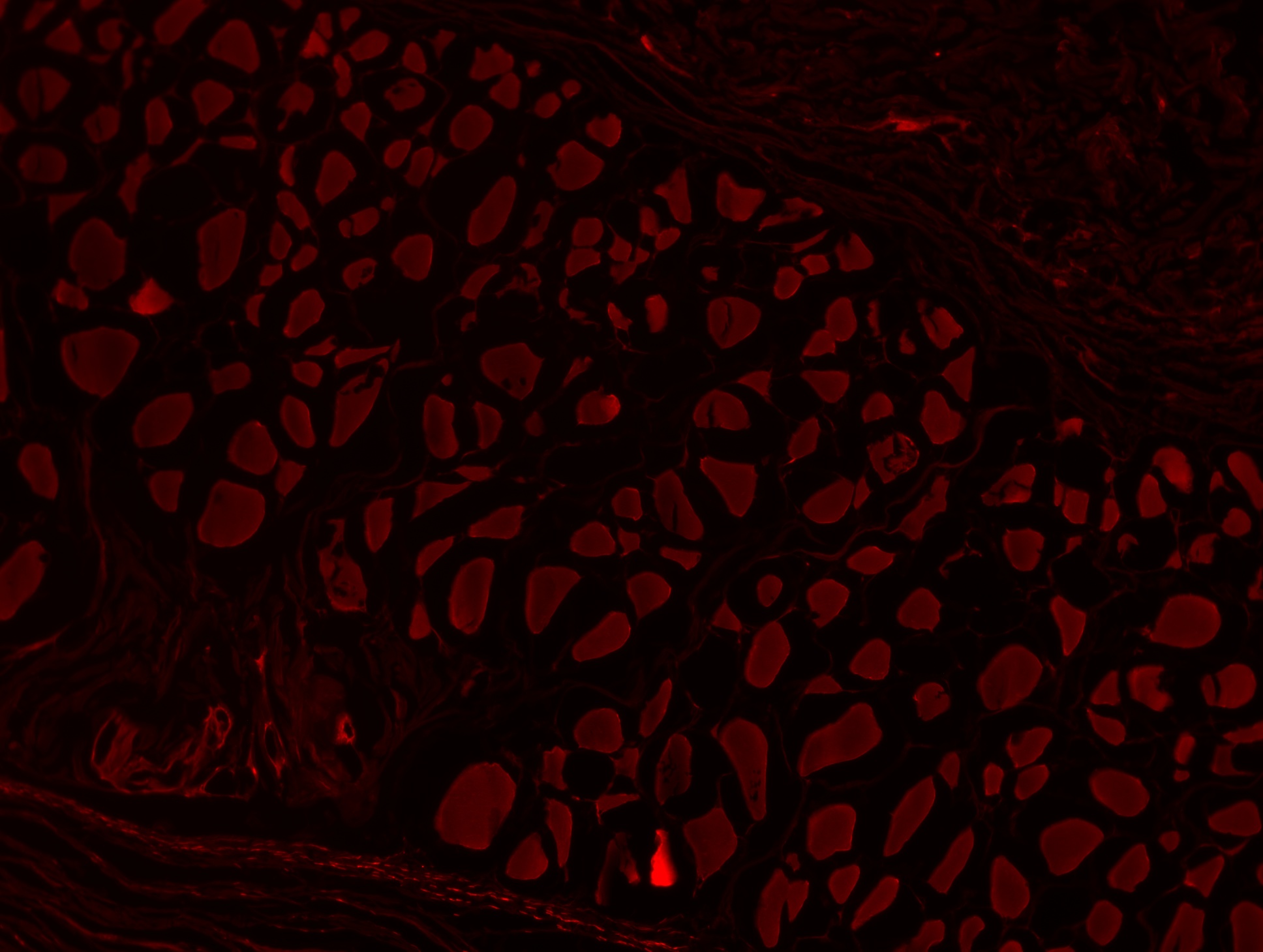

Supplement: S3 File — (ZIP) [file pone.0291080.s003.zip › FIGURE 5 & Raw data/Individual Figure 5 images/Rh-TPGS Transferosomes 1h.jpg]

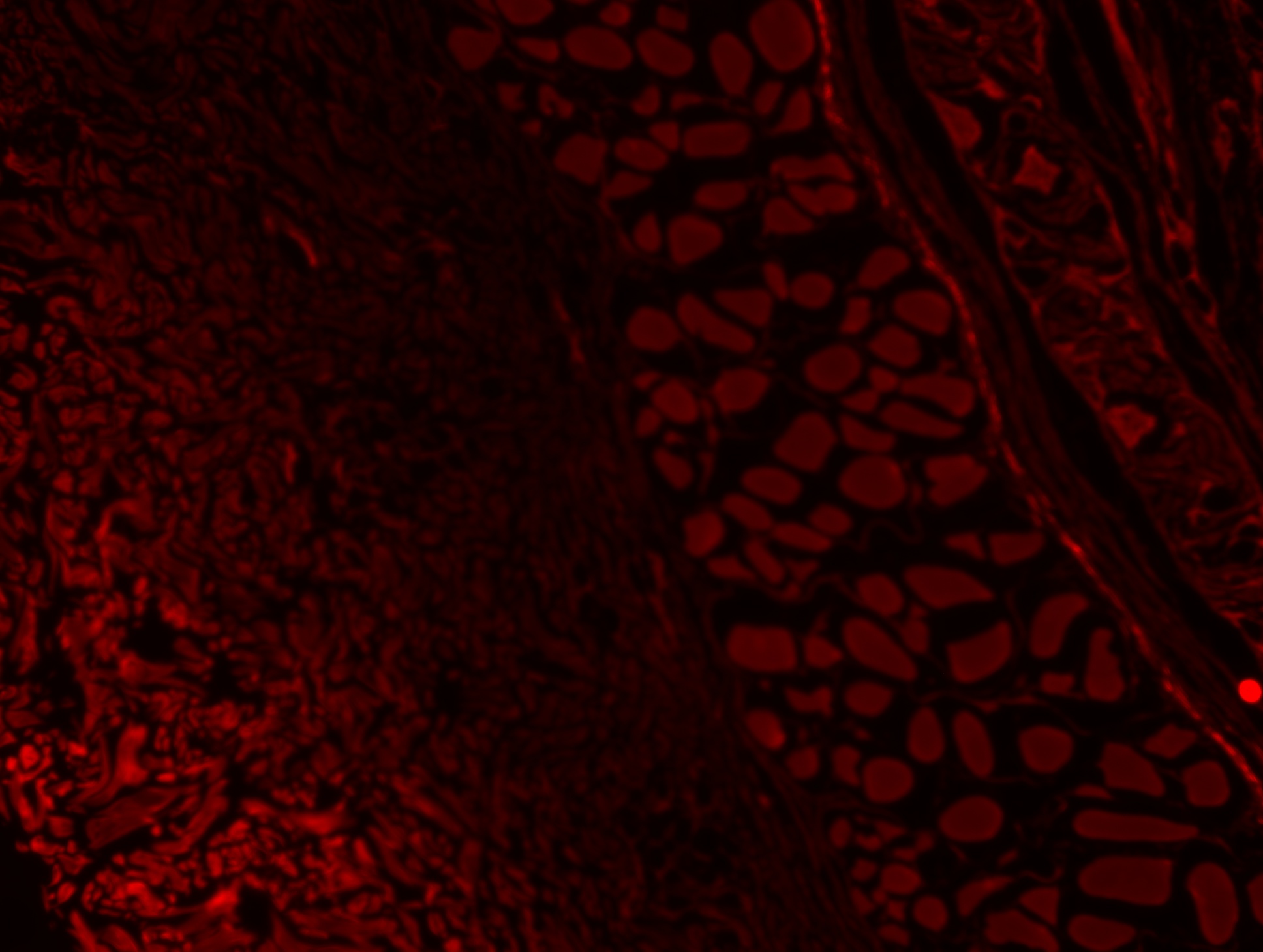

Supplement: S3 File — (ZIP) [file pone.0291080.s003.zip › FIGURE 5 & Raw data/Individual Figure 5 images/Rh-TPGS Transferosomes 4h.jpg]

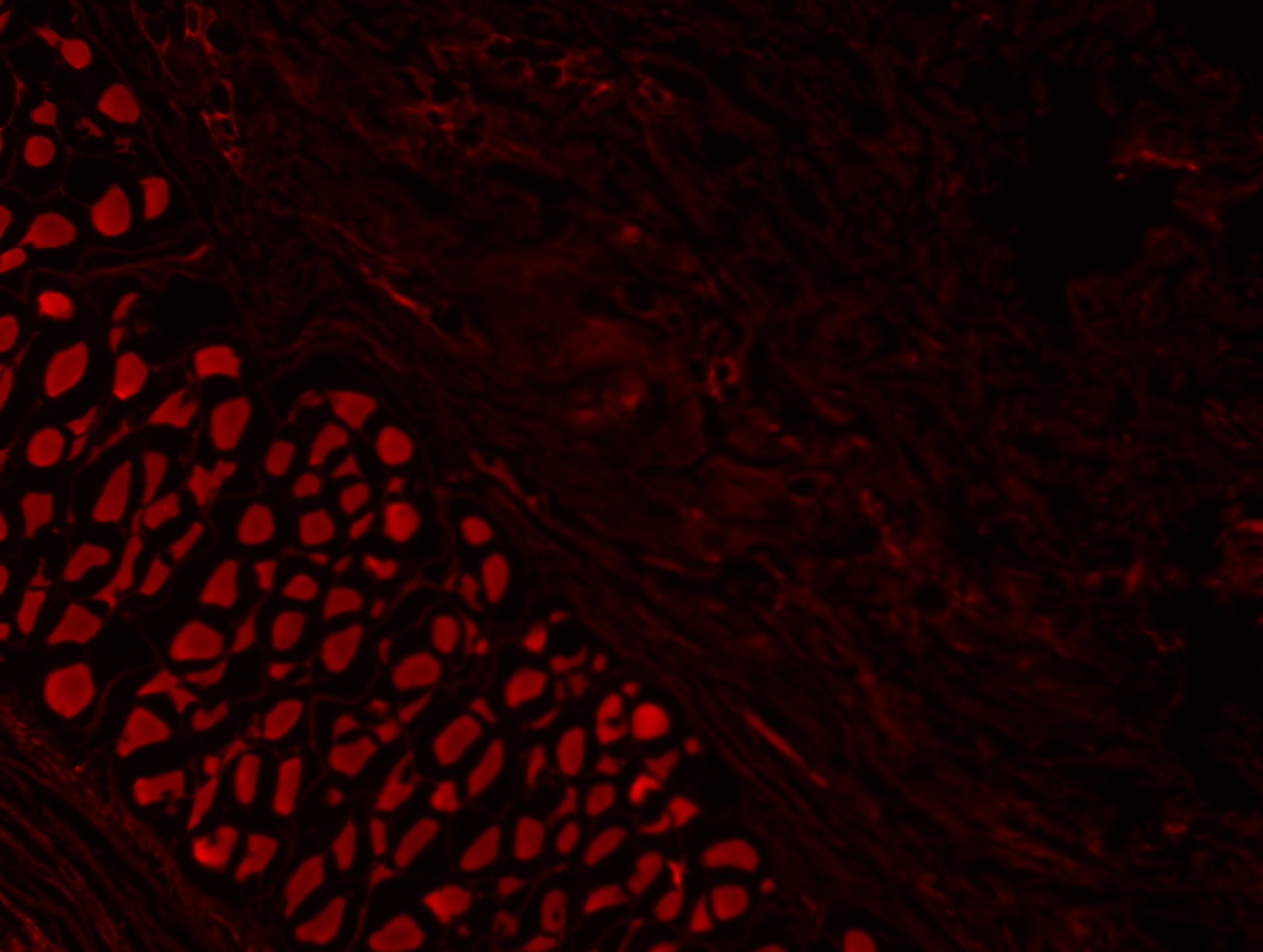

Supplement: S3 File — (ZIP) [file pone.0291080.s003.zip › FIGURE 5 & Raw data/Individual Figure 5 images/Rh-TPGS Transferosomes TAT 1h.jpg]

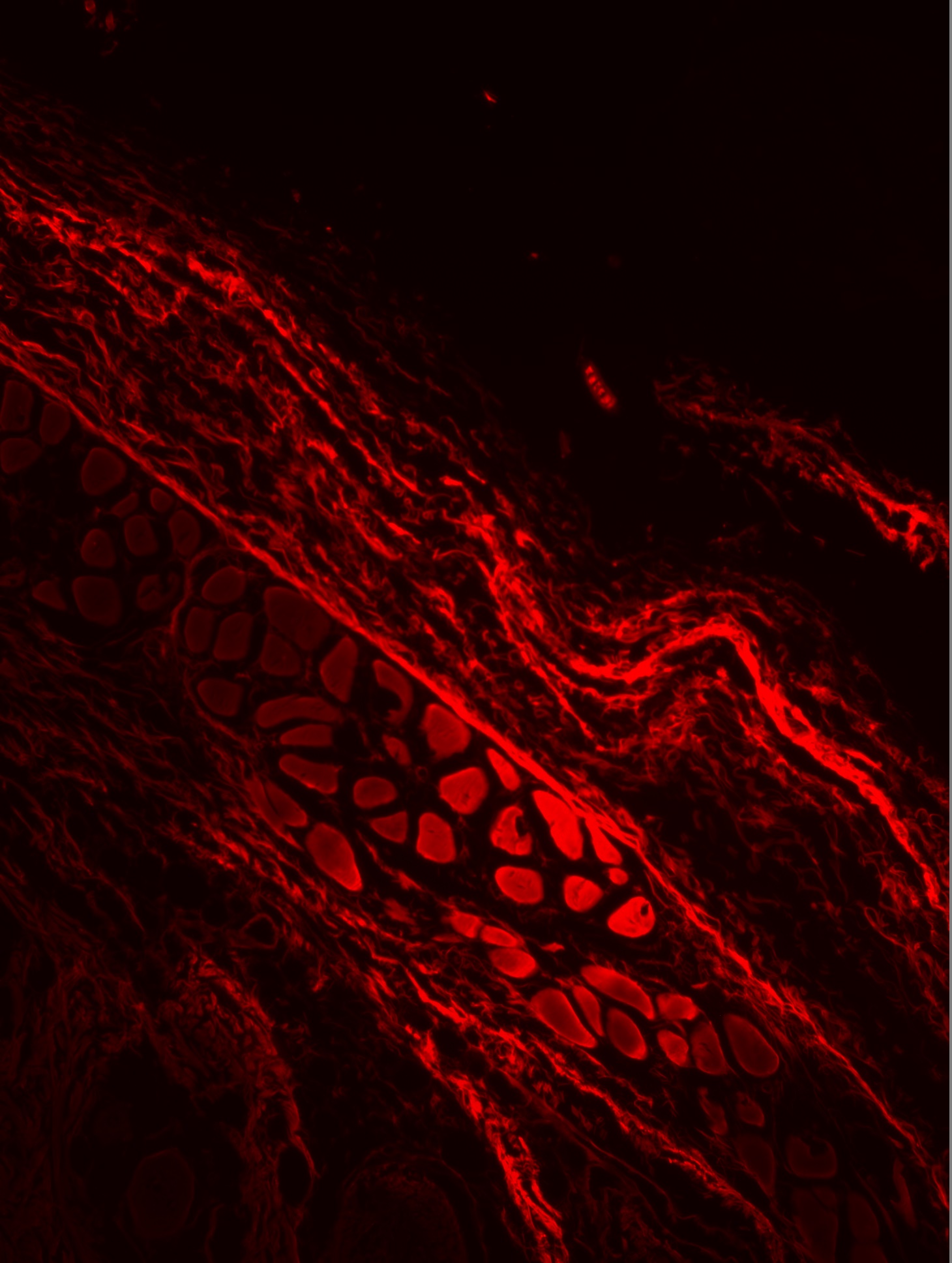

Supplement: S3 File — (ZIP) [file pone.0291080.s003.zip › FIGURE 5 & Raw data/Individual Figure 5 images/Rh-TPGS Transferosomes TAT 4h.jpg]

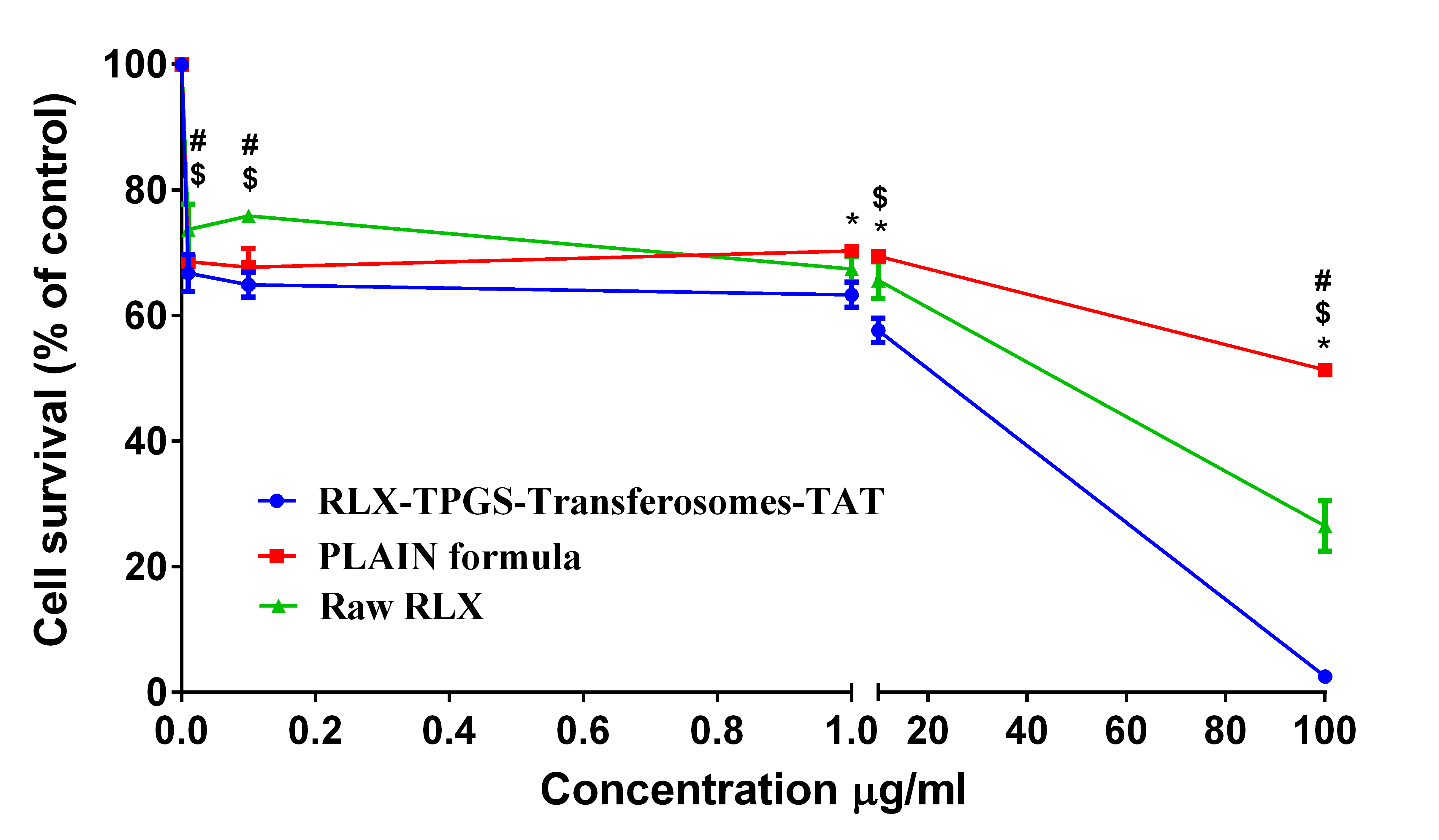

Supplement: S4 File — (ZIP) [file pone.0291080.s004.zip › Figure 6/Figure 6 IC50 RLX.jpg]
