# Supplementary material for: Expression of Concern: Vitamin E TPGS based transferosomes augmented TAT as a promising delivery system for improved transdermal delivery of raloxifene
Source: PLoS One. 2023 Aug 30;18(8):e0291080. doi: 10.1371/journal.pone.0291080 (PMC10468055; doi:10.1371/journal.pone.0291080)
Supplement: S3 File — (ZIP) [file pone.0291080.s003.zip › FIGURE 5 & Raw data/Figure 5 ALL Images from instrument/ALL Rh-TPGS Transferosomes 1h.pptx]

## Slide 1
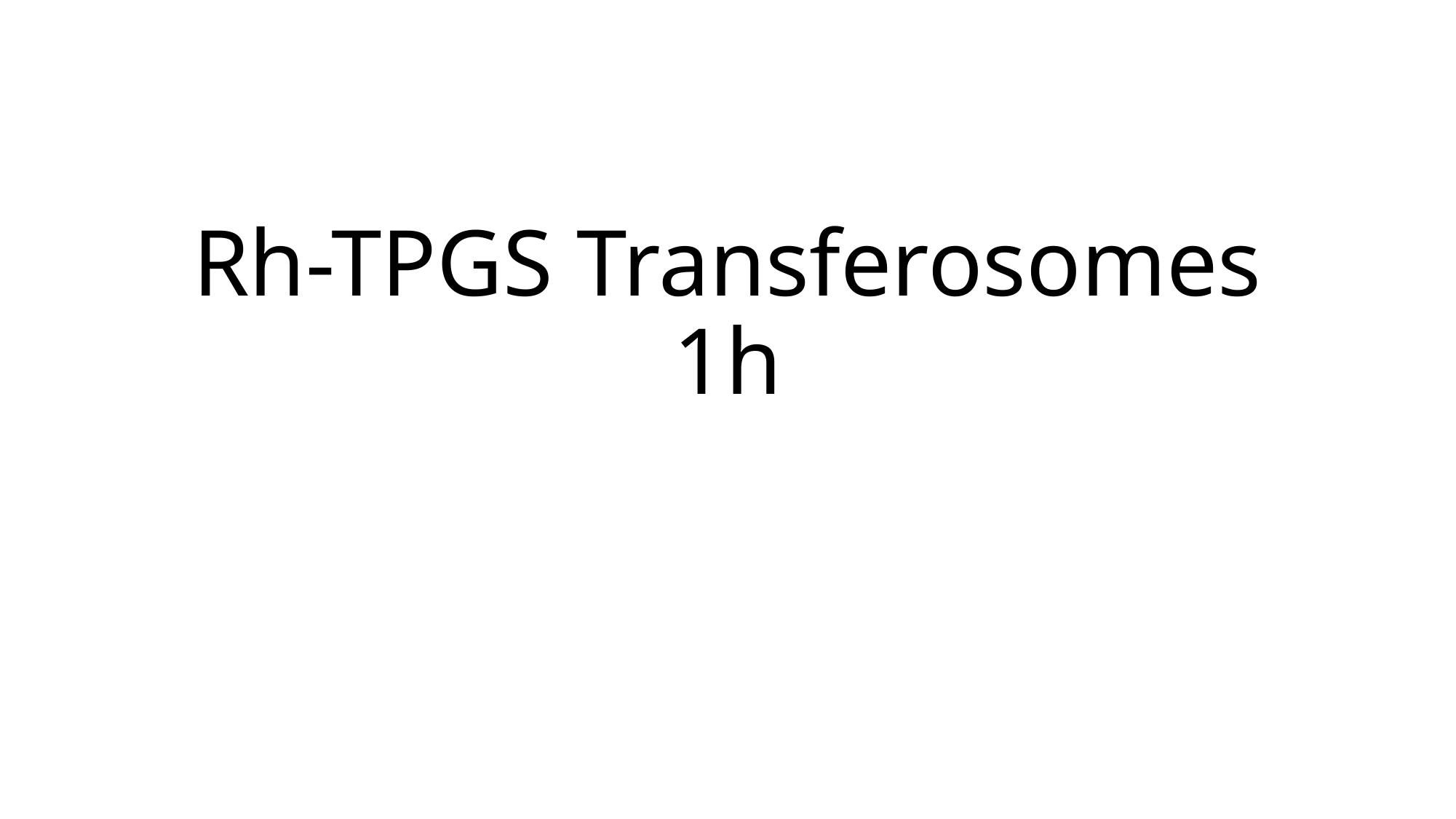

# Rh-TPGS Transferosomes 1h

## Slide 2
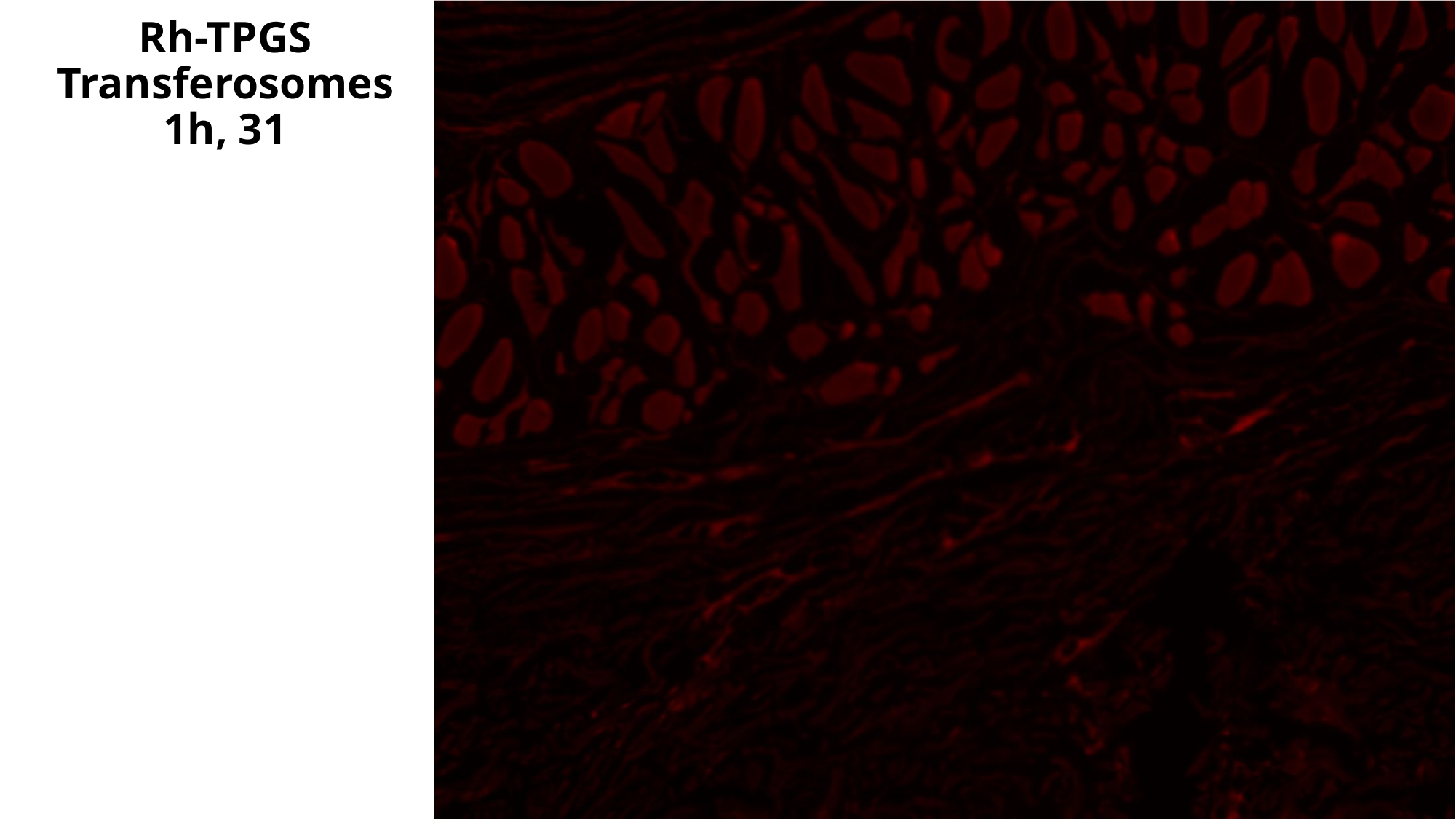

Rh-TPGS Transferosomes 1h, 31

## Slide 3
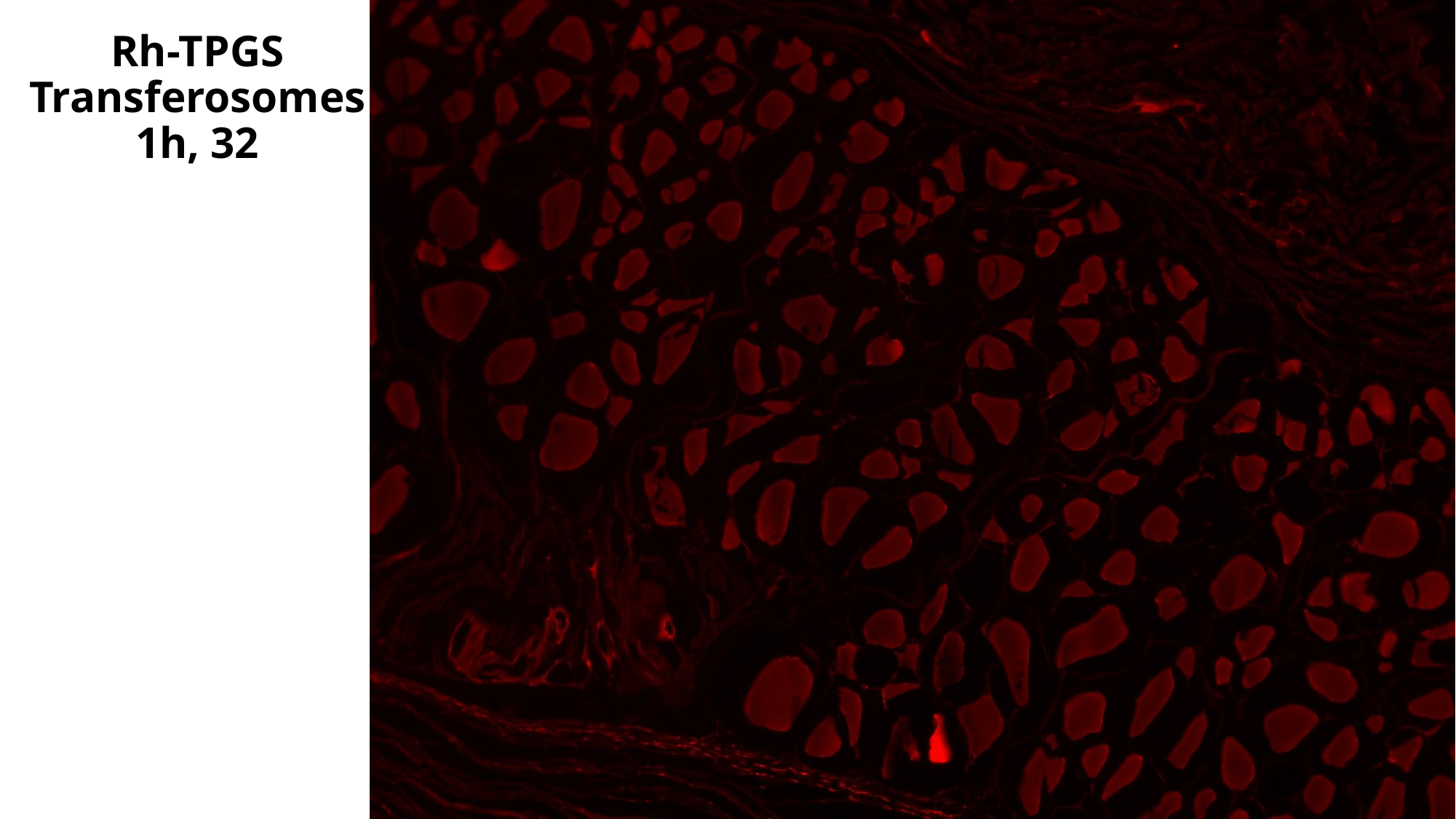

Rh-TPGS Transferosomes 1h, 32

## Slide 4
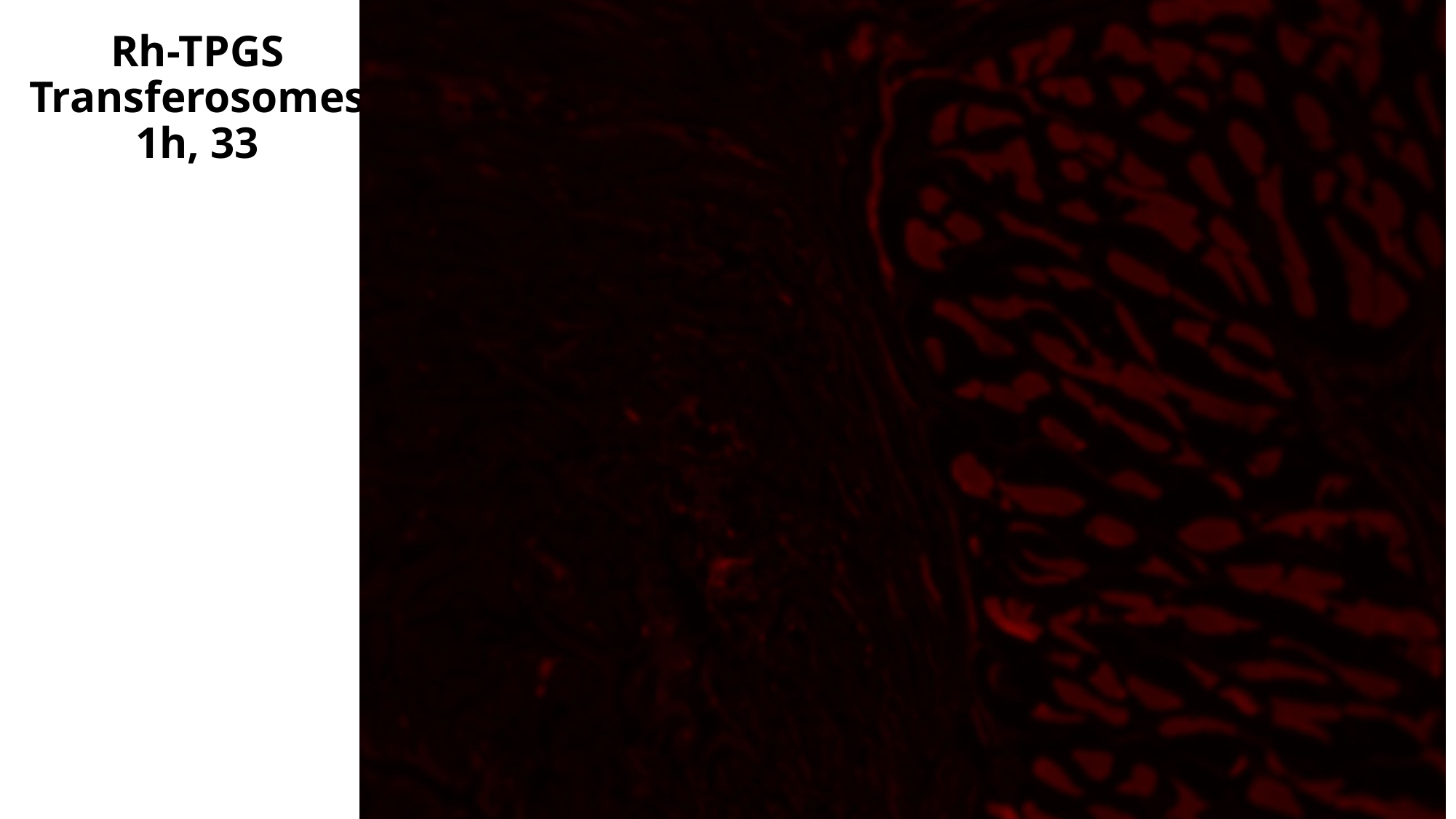

Rh-TPGS Transferosomes 1h, 33
